# Optimization strategies and advances in the research and development of AAV‐based gene therapy to deliver large transgenes

**DOI:** 10.1002/ctm2.1607

**Published:** 2024-03-15

**Authors:** Valeria V. Kolesnik, Ruslan F. Nurtdinov, Ezekiel Sola Oloruntimehin, Alexander V. Karabelsky, Alexander S. Malogolovkin

**Affiliations:** ^1^ Martsinovsky Institute of Medical Parasitology Tropical and Vector‐Borne Diseases, Sechenov University Moscow Russia; ^2^ Center for Translational Medicine Sirius University of Science and Technology Sochi Russia

**Keywords:** exons remodelling, gene editing, gene therapies, inteins, minigenes, protein design, rare diseases, trans‐splicing, viral deliveries, viral vectors

## Abstract

Adeno‐associated virus (AAV)‐based therapies are recognized as one of the most potent next‐generation treatments for inherited and genetic diseases. However, several biological and technological aspects of AAV vectors remain a critical issue for their widespread clinical application. Among them, the limited capacity of the AAV genome significantly hinders the development of AAV‐based gene therapy. In this context, genetically modified transgenes compatible with AAV are opening up new opportunities for unlimited gene therapies for many genetic disorders. Recent advances in *de novo* protein design and remodelling are paving the way for new, more efficient and targeted gene therapeutics. Using computational and genetic tools, AAV expression cassette and transgenic DNA can be split, miniaturized, shuffled or created from scratch to mediate efficient gene transfer into targeted cells. In this review, we highlight recent advances in AAV‐based gene therapy with a focus on its use in translational research. We summarize recent research and development in gene therapy, with an emphasis on large transgenes (>4.8 kb) and optimizing strategies applied by biomedical companies in the research pipeline. We critically discuss the prospects for AAV‐based treatment and some emerging challenges. We anticipate that the continued development of novel computational tools will lead to rapid advances in basic gene therapy research and translational studies.

## Graphical abstract

- AAV‐based gene therapy are on increasing demand to cover the needs in the patient communities with inherited genetic disorders.
- Single AAV vectors delivering functional gene copy are the most popular approach in RnD pipeline.
- Multiple split vectors, inteins and trans‐splicing strategy so far underperform monovector AAV‐delivery.
- Neural networks guided by protein fitness data facilitate *de novo* protein design to fit into AAV capsid.

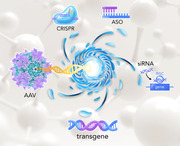

## 1 INTRODUCTION

Recent advances in gene therapy approaches have revolutionized the way to treat inherited genetic disorders, cancer and autoimmune pathologies.^1^, ^2^ The ability to restore the activity of malfunctioned genes is a key paradigm of gene therapeutics. The effector molecules for genetic manipulation (DNA or RNA sequences [e.g. siRNA, miRNA, shRNA, g(guide) RNA]) could be used in a naked state or more efficiently delivered in target cells and tissue using viral or non‐viral (e.g. liposomes, microvesicles and nanoparticles) vehicles.^3^, ^4^ Among the genetic carriers for gene delivery, viral vectors occupy a dominant segment. Viruses possess unique modular characteristics that are suitable for (i) large‐scale production; (ii) flexible genetic manipulation; (iii) high‐throughput analytical methods and (iv) tunable cell or tissue‐specific tropism.^5^, ^6^ In the meantime, non‐viral carriers have also progressed from simply structured nanoparticles to tissue‐specific targeted vehicles with programmable loading capacities.^7^ Technological breakthroughs for non‐viral deliveries make them an attractive platform for large‐scale manufacturing and applications.^8^ For a more detailed analysis of the advantages and limitations of viral and non‐viral vectors, we recommend some comprehensive reviews.^3^, ^9^, ^10^, ^11^

In recent years, the Food and Drug Administration (FDA)‐approved adeno‐associated virus (AAV) has become a blockbuster in gene therapy. AAV's structural simplicity, safety profile and versatility for molecular manipulation have made AAV vectors one of the most popular vehicles for gene delivery.^12^

AAV is a small (25 nm) non‐enveloped virus with an icosahedral capsid carrying a single‐stranded ∼4.7–4.8 long DNA genome that can be either plus or minus polarity.^13^ AAV genome encodes *rep* (Rep78, Rep68, Rep52 and Rep40) and *cap* (VP1, VP2 and VP3) proteins essential for virus replication, genome packaging and capsid assembly.^14^ AAV belongs to the genus *Dependoparvovirus*, Parvoviridae family, and is discovered in various vertebrate species without associations with any pathologies.^15^ Several naturally occurring AAV serotypes have been identified that differ in tissue and cell tropism.^16^ To enhance gene delivery by AAV and minimize ‘off‐target’ transduction, multiple strategies have been employed to generate novel chimeric or synthetic AAV serotypes.^17^, ^18^ The wild‐type AAV life cycle is entirely dependent on the replication machinery of a helper virus, such as adenovirus or herpes simplex virus. The gene therapy platform is based exclusively on the recombinant AAV that encapsidates only expression cassettes devoid of viral rep and cap genes. Trans delivery of viral rep and cap genes enhanced the safety profile of rAAV and minimized potential viral genome integration in host DNA.^19^

The number of clinical trials of AAV‐based gene therapies is skyrocketing, exceeding 200 in 2022. AAV has been successfully used to deliver protein‐coding sequences,^17^, ^20^ antibodies,^21^, ^22^, ^23^ shRNA,^12^, ^20^, ^24^, ^25^ siRNA,^26^ editing tools^27^, ^28^ and anti‐sense oligos.^29^, ^30^ Up to date, AAV‐based gene replacement approaches have been granted approval from US FDA to treat Leber congenital amaurosis (LCA) (Luxturna), haemophilia A (ROCTAVIAN),^31^ haemophilia B (Hemgenix),^32^ spinal muscular atrophy (SMA) (Zolgensma), Duchenne muscular dystrophy (DMD) (Elevidys)^33^ and from European Medical Agency (EMA) to treat adult patients diagnosed with familial lipoprotein lipase deficiency (LPLD) (Glybera) (authorization has expired).^25^

Despite its tremendous popularity in clinical trials and basic research, AAV suffers from a notoriously limited packaging capacity. Due to its small capsid size (250 A in diameter), only genomes up to ∼4.8 kb can be efficiently packaged and produced. This inconvenient drawback significantly hinders the wide use of AAV vectors in gene delivery therapies, where the size of the transgene may exceed the available capsid inner space. Some studies suggest that different AAV serotypes can encapsidate nucleotide sequences larger than 4.8 kb, but the exact molecular mechanism of this phenomenon is not well described.^34^, ^35^, ^36^ Individual reports also suggest that AAV's close relative, human parvovirus‐bocavirus, has a larger capsid interior and can accommodate bigger DNA (>4.8 kb).^37^, ^38^

Considering AAV vectors as an indispensable tool for gene delivery several elegant approaches have been developed to circumvent the limited capsid capacity. In this review, we describe the solutions and strategies that have been proposed and/or used to mediate the transfer of larger transgenes using AAV vectors. To unravel the diversity of AAV‐based approaches, we searched for relevant information from registered clinical trials (clinicaltrials.com), company websites, press releases, patents and any open‐source announcements. We also searched PubMed for research articles, meta‐analyses and systematic reviews describing AAV‐based gene therapy, using the search terms (‘AAV’ OR ‘Adeno‐associated virus’ OR ‘gene therapeutics’ OR ‘Viral vector’) AND ‘gene therapy’, without language restrictions. As a further matter, we focused our search on the translational use of recent advances in AAV‐mediated gene therapy used by pharmaceutical companies.

Here, we review progress in the development of minimally functional copies of transgenes, the so‐called minigenes, using rational design and protein modelling approaches. The key parameters and optimizing strategies of AAV expression cassettes are discussed. In addition, we summarize the results of AAV‐based gene therapies in research and development (RnD) pipelines using multiple AAV vectors carrying split transgenes, trans‐splicing and exon‐skipping approaches with particular emphasis on clinical applications. We resume the perspectives of neural networks and computational approaches to design short and functional transgenes compatible with AAV vectors.

## 2 MINIGENES – MINIMAL FUNCTIONAL GENE VARIANTS

Multiple protein‐encoding sequences from human and animal genomes exceed AAV vector packaging capacity. Importantly, to make AAV vectors functional, gene‐coding sequences should be accompanied by expressing machinery (i.e. AAV inverted terminal repeat sequences [ITRs], promoter, poly(A) signal) to mediate gene transfer and long‐term expression. These requirements additionally shorten potential transgene size for AAV gene therapy. The empirically defined limit of an efficiently packaged genome into an AAV capsid spans around 4.8 kb. The current consensus is that rAAV optimally accommodates transgenes that are up to 3.5 kb.

**FIGURE 1:**
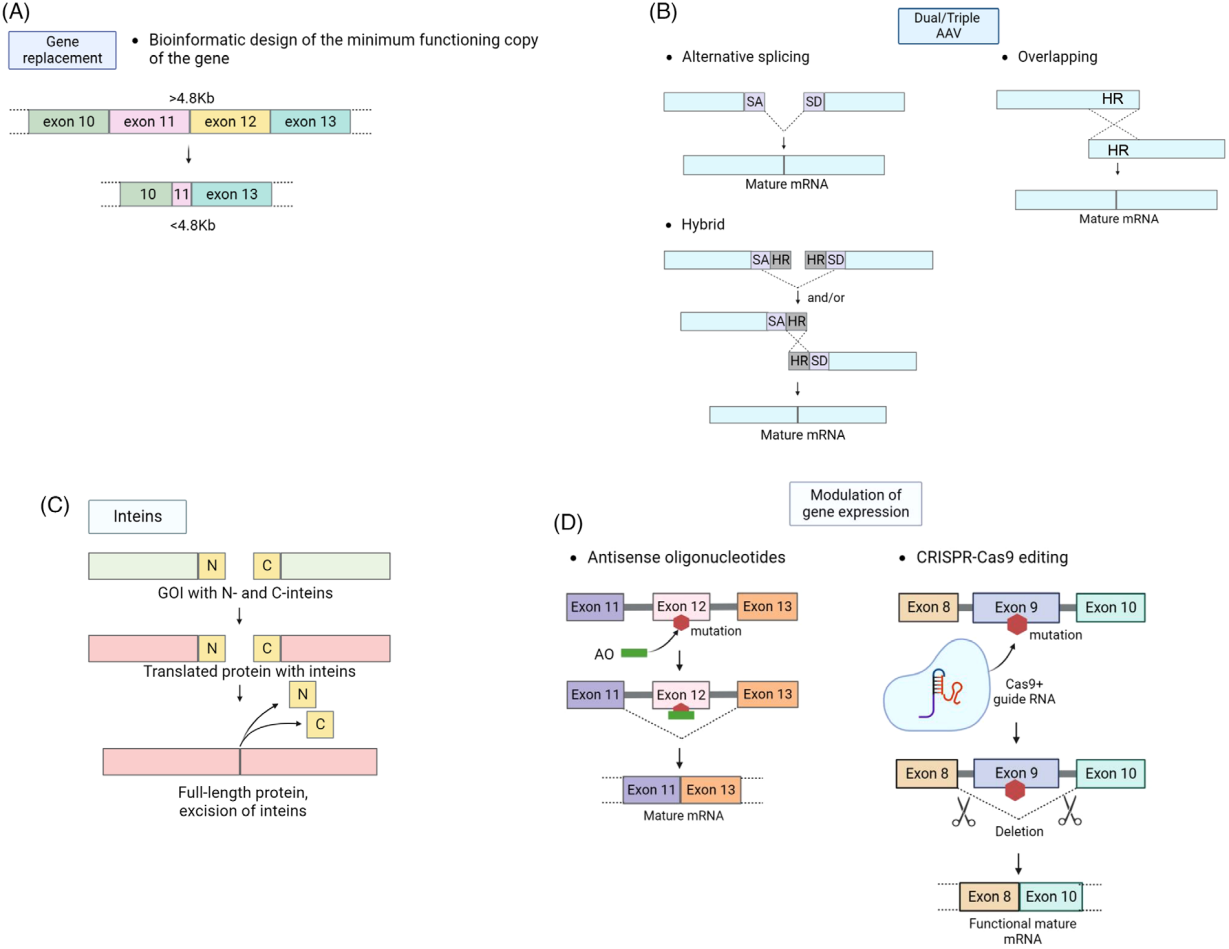
Schematic representation of bioengineering approaches for adeno‐associated virus (AAV)‐based gene therapy targeting big genes. Big transgenes exceeding AAV vector packaging capacity (>4.8 kb) can be redesigned as minigenes (A) or split in AAV vectors using trans‐splicing, hybrid approaches (B) or inteins (C). In other cases when the expression of big transgene should be regulated, antisense oligonucleotide (ASO)‐based therapy is applied (D). Gene editing using AAV vectors carrying ISPR‐Cas9 tools also can be used to exclude mutated exons from DNA sequence and provide a template for following alternative splicing and mature mRNA formation (D). C, carboxyl terminus of a protein; CRISPR, Cas9 clustered regularly interspaced short palindromic repeats and CRISPR‐associated protein 9; HR, highly recombinogenic sequence; N, amino terminus of a protein; SA, splice acceptor site; SD, splice donor site.

This boundary pushes forward the progress towards the design of novel shorter gene variants while maintaining their function. There is no blueprint for designing minimal functioning copies of the genes available elsewhere. Here, we highlighted some tools and main steps to design desired protein, depending on the targeted protein structure (Figure 1).

The main pillar for minigene design is structural bioinformatics methods and rational design involving protein and gene databases. Available and well‐defined 3D structures of protein of interest significantly facilitate protein engineering. In addition, the reconstruction of proteins accompanied by molecular dynamics simulations helps to screen out thousands of unfitting variants and narrow down the experimental pipeline.^39^

Several minigenes compatible with AAV vectors’ packaging capacity have been rationally designed and functionally tested. Mini‐otoferlins (*OTOF*) partially restored the physiological functions of AAV8 transduced auditory hair cells of OTOF knock‐out mice.^40^ The AAV9‐PHP.B was used to transfer mini‐versions of the protocadherin‐15 (*mini‐PCDH15*) to rescue hearing loss in the Myo15‐Cre conditional knock‐out mice showing great potential as a future gene therapy for inherited deafness (Usher syndrome type 1 F).^41^ Similarly, the minigene‐4 is a shortened variant of the *USH2A* gene that was proposed for AAV‐mediated gene therapy for hereditary vision and hearing loss – Usher syndrome type 2.^42^ Another rational protein miniaturization was performed with cilia‐centrosomal protein encoded by *CEP290* gene mutations which are frequently associated with autosomal recessive childhood blindness disorder LCA. MiniCEP290 gene was designed and delivered by the AAV2/8 vector and showed a delay in retinal degeneration in the Cep290^rd16^ mice.^40^ The compact form of tuberin encoded by the ‘condensed’ с*TSC2* gene was proposed for gene therapy of tuberous sclerosis complex.

A vivid example of the successful design of a minimal functioning copy of a gene is the long‐standing research of multiple laboratories supported by biomedtech companies to develop the dystrophin minigene (microdystrophin) for DMD therapy.

Dystrophin is a humongous protein consisting of 79 exons and encoded by >2200 kb gene (protein‐coding sequence 11.05 kb).^43^ The DMD gene encodes a cytoskeletal protein dystrophin, and its main function is acting as a tether connecting intracellular actin filaments to the sarcolemma membrane.^43^, ^44^ Deletion, duplication and point mutations in hot spot exons lead to severe and progressive muscle fibre degeneration and weakness. Importantly, DMD is a result of out‐of‐frame mutations in the dystrophin gene that cause premature stop codon and as a result block dystrophin protein translation. Because of its size, the dystrophin gene for a long time remained an unattainable target for gene therapy. Intriguingly, a similar hereditary neuromuscular pathology called Becker muscular dystrophy (BMD) is caused by defective truncated dystrophin, which is only 46% of the full‐length protein. However, patients with BMD present moderate muscle weakness and are still able to walk at older age.^43^, ^44^, ^45^ This fact indicates a fairly mild progression of the disease. It was concluded that even a truncated version of dystrophin can restore protein functionality and this phenomenon could be used to create short and functional genetic therapeutics for patients with DMD. Since then, multiple studies have shown that miniaturized DMD gene can be efficiently packaged into AAV capsid with muscle cell‐specific promoters and delivered to targeted tissue.^20^, ^46^, ^47^ The recent approval of Elevidys for DMD therapy has demonstrated that a minigene approach is a viable and potent solution for AAV‐based drugs.

A similar minigene strategy has been applied for 2023 marketed *Valoctocogene roxaparvovec* (ROCTAVIAN) from BioMarin Pharmaceutical Inc. for haemophilia A treatment. ROCTAVIAN is an AAV5‐based therapeutics carrying human Factor VIII (FVIII) driven by tissue‐specific promoters to liver cells. FVIII is a large plasma glycoprotein of 2332 amino acid residues organized in six domains: A1‐A2‐B‐A3‐C1‐C2.^48^ The size of the FVIII gene (7.05 kb) exceeds the capacity of the AAV. Notably, the B domain occupies ∼44% of FVIII, and mutations in it are responsible for 15%–26% of severe haemophilia A. Interestingly, the B domain has no known homologues and FVIII exerting coagulation function does not possess B domain.^49^

Most recently, FVIII, lacking most of the B domain or entire FVIII‐B, has been raised as a therapeutic candidate for AAV therapy. In addition to ROCTAVIAN, Opti‐Dys.delta.3978 from Pfizer Inc. is in Stage III of clinical trials, 2 drugs for the treatment of haemophilia A from Takeda/Spark Therapeutics are in stage II, and 1 drug from Takeda is in stage I/II of clinical trials (Table S1).

## 3 MODIFICATIONS OF GENETIC REGULATORY ELEMENTS OF AAV EXPRESSION CASSETTES

**FIGURE 2:**
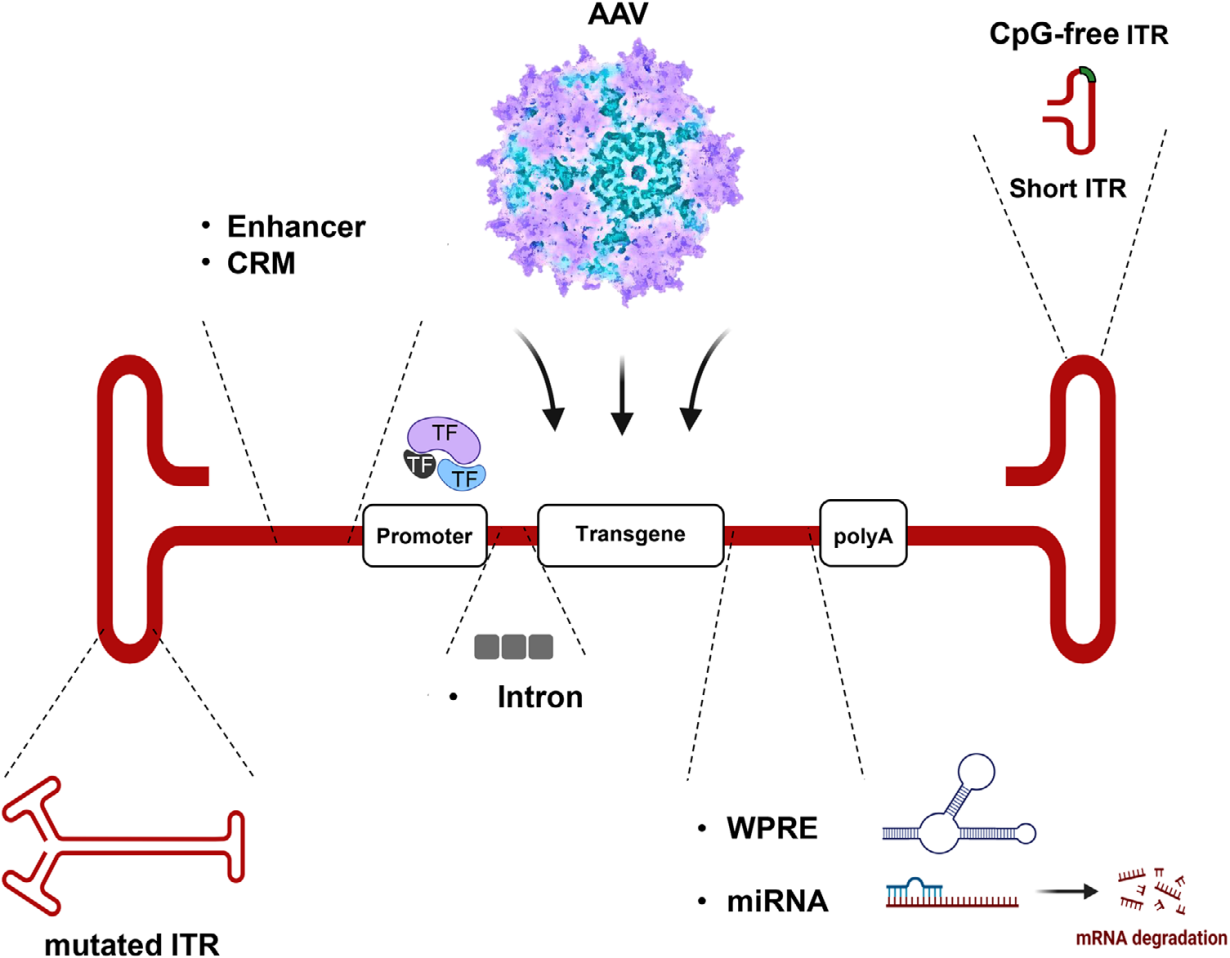
Adeno‐associated virus (AAV) expression cassette elements and modifications. Critical elements of the AAV expression cassette are promoter, transgene and poly(A) sequence and inverted terminal repeat (ITR)’s flanking virus genome. Additional elements such as CRM, enhancers, intron, WPRE, miRNA and others may enhance AAV transduction and modulate transgene expression. All elements of the AAV expression cassette can be genetically modified and optimized. CpG, regions of DNA where a cytosine nucleotide is followed by a guanine; CRM, cis regulatory module; miRNA, micro RNA; mRNA, messenger RNA; TF, transcription factors; WPRE, woodchuck hepatitis virus (WHV) posttranscriptional regulatory element (WPRE).

Besides the size of a transgene, several biological aspects of AAV vectors shape their development and application of gene therapeutics. Genetic regulatory elements of the AAV expression cassette, regardless of the AAV serotype and capsid structure, such as ITR, enhancer, promoter, introns, posttranscriptional regulatory elements (PRE) and poly(A) signal, directly affect vector capacity, transduction efficiency and transgene expression profile.^50^ Four key elements of AAV expression cassettes are essential for successful gene transfer and expression (ITRs, promoter, transgene and polyA signal) (Figure 2). All other accessory regulatory compounds may significantly enhance or modulate the expression profiles of transgenes if AAV vector capacity allows. In this subsection, we briefly review some critical aspects of ITRs, promoters and polyA signal design and their biological significance. In additional details of AAV genetic engineering and expression cassettes optimization, readers may find in recent reviews.^51^, ^52^, ^53^

Core elements of typical AAV expression cassettes are promoters. Promoters are cis‐acting regulatory elements that drive, regulate and enable transcription of the transgene (s) that they are linked with. Transgene transcription mediated by RNA polymerase II is strictly defined by the promoter accompanied by various cell‐specific transcription factors. The length of the promoters varies from ∼100 to −1500 nucleotides and may significantly decrease the capacity of AAV vectors. Constitutive ubiquitous and strong promoters are often used to control the expression of any transgenes in AAV cassettes. The most popular promoters in AAV expression cassettes are human cytomegalovirus (hCMV), the short CMV early enhancer/chicken β actin (sCAG), mouse phosphoglycerate kinase (mPGK) and human synapsin (hSYN) promoter, the human polypeptide chain elongation factor (EF1α) and the ubiquitin C (UbiC).^50^, ^54^, ^55^

Nevertheless, constitutive promoters provide a high expression level of a transgene that is not always needed in physiological conditions. Moreover, overexpression of AAV‐delivered cargo may trigger an immune response to the transgene that may eventually halt its functionality. Tissue‐specific promoters in this case are viable and safe alternatives to minimize potential adverse effects of AAV gene therapy. For instance, various muscle‐specific promoters (e.g. creatine kinase promoters (CK6, CK8 and MHCK7)), desmin promoters, human α‐myosin heavy chain gene (αMHC) promoter, the myosin light‐chain promoter (MLC2v) and the cardiac troponin T promoter (cTnT) are attractive options for AAV‐based therapeutics to treat inherited muscular dystrophies (i.e. SMA, DMD and Pompe disease).^56^

Liver‐specific promoter (LP1) was successfully used to develop AAV‐based therapeutics to treat factor IX (FIX) deficiency in haemophilia B patients (Hemgenix).^57^, ^58^ Another hybrid LP was implemented in an AAV expression cassette delivering B domain depleted human FVIII gene (ROCTAVIAN).

Several small, the so‐called micro‐promoters, only 84 (MP‐84) and 135 bp (MP‐135) in length were designed and demonstrated robust activity in cell lines from different germ layers.^59^ The sequences of MP‐84 and MP‐135 promoters originate from the human insulin and glucagon promoters regions respectively and demonstrated comparable activity with much larger CAG promoter in human islet endocrine cells, hepatocytes, brain and muscle tissues.^59^ Short promoters may be helpful to accommodate large transgene in AAV expression cassettes and include additional regulatory elements (i.e. enhancers, introns and miRNA).

Current advances in genetic engineering allow to design and synthesize of any desirable promoter that could be used in AAV expression cassettes. Due to the well‐known modular structure of promoters, they can be used to produce personalized AAV expression vectors to treat inherited disorders.

Finally, the role of the promoters in the AAV expression cassette is invaluable, and supposedly, the ideal promoter for AAV‐based gene therapy should: (i) be short in length (∼300 bp); (ii) cell or tissue‐specific; (iii) provide physiological transgene expression level; (iv) be switchable or tunable and (v) non‐toxic or immune tolerant.

AAV ITRs (palindromic ∼125(+20) bp sequence) are genuine replication and packaging signals that also promote long‐term genome persistence and as a result prolonged transgene expression. The canonical T‐shaped hairpin loop of AAV ITRs is essential for AAV Rep protein binding and initiation of AAV genome concatemerization.^60^ Most of the current AAV‐based therapeutics rely on the AAV2 ITRs; however, other AAV serotype‐specific ITRs have also been explored.^61^

Modifications of AAV ITRs have been applied to generate self‐complementary AAV vectors (scAAV) with increased transduction efficiency and transgene expression.^62^, ^63^ scAAV expression cassettes have mutated ITRs and double‐stranded DNA genome (Figure 2) that significantly speed up AAV genome replication and transgene transcription as the requirement for complementary‐strand synthesis is satisfied and DNA has already folded into transcriptionally active double‐stranded form through intra‐molecular annealing.^64^ Several studies indicate an increased efficiency of transduction from scAAV vectors over conventional rAAV.^64^, ^65^, ^66^

Shortened AAV ITRs with an 11–14 bp deletion can also be used to produce functional AAV vectors.^67^ Two ITRs flanking the AAV genome have a unique ability of self‐correction and tolerant relatively large deletions; however, direct modification of structurally important regions within ITRs (D‐sequence, Rep protein binding element, terminal resolution site [trs]) may significantly reduce Rep binding activity and as a result AAV vector assembly.^68^ For instance, modifications of the trs 7‐bp core sequence decreased Rep protein nicking activity by 20%–50% compared to the wild‐type ITRs.^15^

Despite the aforementioned advantages, scAAVs have a decreased capacity to ∼2.5 kb compared to conventional single‐stranded AAV that drastically limits their wide application.^65^, ^69^

Other components of AAV expression cassettes such as PRE and poly(A) signals are also objects of optimization to expand the capacity of AAV vectors. The SV40 virus, human growth hormone and bovine growth hormone polyadenylation signals are the most commonly used in AAV expression cassettes.^70^, ^71^ The selection of the poly(A) signal is often defined by the transgene size; however, the shortened poly(A) sequence does not always fully recapitulate the full‐size analogue. A variety of shortened AAV expression cassettes were generated by Choi et al.^72^ Focusing on the Woodchuck hepatitis virus PREs and poly(A) elements, the authors declared 399 bp reduction in the CW3SL AAV vector without losing EGFP expression level in AAV transduced DIV9 cultured hippocampal neurons relative to the full‐length CWB cassette. Moreover, the level of green fluorescent protein (GFP) expression delivered by the CW3SL vector in the hippocampal CA1 region of mice was similar (86.2%) to the parental CWB AAV expression cassette.^72^

Therefore, the optimization of the AAV expression cassettes may also significantly increase AAV packaging capacity, modulate transgene expression profile and enhance transgene expression.

## 4 EXON SKIPPING

Exon skipping is a molecular strategy aimed at removing specific exons, frequently associated with pathological mutations in a gene of interest. Exon skipping can be initiated by using antisense oligonucleotides (ASOs) or clustered regularly interspaced short palindromic repeats with Cas9 protein (CRISPR/Cas9) targeting exonic or intronic sequences important for RNA splicing. As a result of minimizing the number of exons in full‐length genes, a functional minigene variant is transcribed (Figure 1). The ASO and CRISPR/Cas9 variants can be delivered to the cells using viral vectors including AAV.^73^ Recent research has focused on using CRISPR tools, including Cas12a, Cas12f1 and engineered guide RNAs, to enhance gene delivery by AAV vectors. The development of compact CRISPR systems and the remodelling of guide RNA structures have been shown to enable multiplexed and efficient genome editing when delivered by AAV vectors. These advancements have the potential to overcome the size limitations and AAV vectors and expand their applications in gene therapy.^74^

ASOs (or AON) are short DNA sequences that are complementary to the target gene region. Hybridization of ASO to the selected gene sequence leads to the exon shielding which then becomes inaccessible for splicing machinery. One of the first successful results with exon skipping was generated in 1993, the function of the human beta‐globin gene was replaced in vitro using a 2′O‐methyl RNA ASO.^73^, ^75^ Along with advances in gene synthesis (high‐throughput and low‐cost production), this area has begun to strive rapidly, and nowadays, the large‐scale libraries of antisense oligos targeting different human genes are being developed to treat a wide range of pathologies.^73^, ^75^, ^76^, ^77^, ^78^ We identified 14 products that use either ASO or RNA interference approaches with AAV vectors. The separate list of AAV‐delivered ASO and RNA‐based drugs is summarized in Table S2.

AAV vectors are frequently used for target delivery of CRISPR‐Cas9 systems for gene correction. AAV‐based CRISPR‐Cas9 transfer has been applied to edit disease‐responsible mutations in several preclinical models of human pathologies (DMD, hypercholesterolemia and urea cycle disorders).^79^, ^80^, ^81^ Due to the big size of the CRISPR‐Cas9 components (sgRNA and Cas9) and the limited capacity of AAV capsid, such genetic cargo should be packaged into individual AAVs. Promising data indicate that a dual‐AAV system enables gene editing in mouse models. Yang et al. tried to correct a metabolic liver disease in mice (ornithine transcarbamylase [OTC] deficiency) by intravenously infusing two AAVs, one expressing Cas9 and the other expressing a guide RNA, and the donor OTC DNA sequence.^82^ The authors demonstrated the reversion of disease phenotype in 10% (6.7%–20.1%) of hepatocytes and overall increased mice survival. In conclusion, the authors cautiously address striking differences in clinical outcomes following AAV‐mediated gene correction via homology‐directed repair and worry that it may limit wide applications of therapeutic genome editing.

Recent advances in genome editing methods enable to customize and tune gene editing approaches for maximum specificity, efficiency and tolerability. One of the peculiar examples is a smaller Cas9 orthologue from *Staphylococcus aureus* (SaCas9) that is 1 kb shorter than commonly used SpCas9.^83^, ^84^ Successfully packaged SaCas9 and gRNA into one AAV8 vector demonstrated >40% of Pcsk9 gene modification in mouse liver, significant reduction of serum Pcsk9 and decrease of total cholesterol level.^80^ Although no signs of vector and cargo‐mediated toxicity were observed, long‐lasting studies are necessary to fully assess the safety profile of AAV‐mediated gene correction with either Cas9 orthologue. The plethora of AAV cargos (e.g. CRISPR/Cas9, base editors, shRNA, siRNA and ASO) and its application in gene therapy are briefly mentioned in this review, and we refer readers to comprehensive articles with more examples dedicated to this exciting topic.^19^, ^21^, ^85^

Editas Medicine designed an AAV‐5 vector that delivers staphylococcus Cas9 and CEP290‐specific guide RNA (commercially named EDIT‐101) by subretinal injection for the treatment of LCA 10 (LCA10), by deleting the IVS26 CEP290 mutant allele. The *CEP290* gene, also known as *NPHP6*, is 93.2 kb in length and contains 55 exons. It encodes Centrosomal Protein 290, a 290 kDa protein of 2479 amino acids with multi‐domain coiled‐coil domains, CEP290 found in the centrosome/basal bodies. Mutations in the CEP290 also lead to Joubert syndrome associated with severe congenital blindness or other forms of early retinitis pigmentosa.

Mutation‐specific gRNA recruits Cas9 for targeted region excision, and natural splicing of CEP290 mRNA is initiated. As a result, a functional CEP290 protein is expressed. Maeder et al. have demonstrated the effectiveness of AAV5‐based gene editing of the CEP290 gene in a humanized CEP290 mice model.^86^ Positive initial clinical data from Phase 1/2 BRILLIANCE clinical trial of EDIT‐101 demonstrated clinical proof of concept. Despite pioneering CRISPR in vivo trials, Editas reckoned the efficacy was not strong enough to pursue the development of the therapy on its own and is pausing BRILLIANCE, whereas it looks for a development partner to take the programme forward. It should be pointed out that EDIT‐101 is the first CRISPR‐Cas9 in vivo drug in clinical trials on patients; previously comparable therapy was performed only ex vivo.^87^ It is also worth noting that an appropriate legal framework for in vivo gene editing and the use of CRISPR‐Cas drugs in patients needs to be properly established to address potential ethical issues.

AAV vectors carrying modified *U7snRNA* gene, from which ASO could be transcribed, are alternative therapeutic approaches for traditional AAV gene replacement therapy, especially in a case where the size of a gene exceeds AAV vector packaging capacity. U7snRNA functions as a splicing modulator and together with small nuclear ribonucleoprotein particles shields ASO from degradation.^88^ Thereby, the AAV9 vector expressing U7 small nuclear RNAs targeting *DMD* exon 2 (scAAV9.U7snRNA.ACCA) has been successfully tested in the Dup2 mouse model. The results of in vivo studies demonstrate that a single neonatal injection of scAAV9.U7snRNA.ACCA resulted in highly efficient and long‐term exon skipping, dystrophin production and almost complete correction of the disease phenotype at 6 months.^89^ Similarly, the long‐term efficacy of AAV9‐U7snRNA‐mediated Exon 51 skipping in *mdx52* mice was confirmed with the restoration of dystrophin expression. However, the data indicate that the efficiency of AAV‐mediated exon‐skipping could be dependent on the targeted exon which could potentially limit its wide application.^90^

The exon‐skipping mechanism makes it possible to develop a wide range of mutation‐specific therapeutic agents for a variety of pathologies. Although exon skipping can help restore the reading frame of a gene, it may not fully restore the normal function of the gene. This can result in only partial restoration of targeted gene re‐expression and improvement of the disease phenotype.

Nevertheless, not all genetic pathologies can be successfully treated with ASO or CRISPR‐Cas9 therapy, especially when the mutations are equally distributed throughout the gene. In this case, only complete gene addition therapy is a viable option.

Likewise, ASOs must be selected individually for each patient, depending on the mutation profile and the specific exon that needs to be skipped. Thus, the costs of producing such a type of therapy may be unbearable for every needed patient. For example, Spinraza requires the administration by a lumbar puncture into the cerebrospinal fluid, which is undoubtedly associated with risks for the patient, because of sedation, and requires certain hospital conditions and surgical training. The efficacy of exon skipping can vary among patients, and some patients may not respond to treatment as effectively as others. This variability can make it challenging to predict treatment outcomes.

Common side effects of ASOs‐based drugs are neurotoxicity, thrombocytopenia and blood clotting disorders.^18^

## 5 MULTIPLE AAV VECTORS

Dual or triple AAV vector delivery is a technology that allows the division of cDNA fragments into multiple parts and encoding each into its own AAV vector, thus having advantages in capacity over a single AAV. This technology was developed due to the properties of the AAV genome to be concatemerized in the head‐to‐tail direction.^86^, ^91^ Dual AAV vectors have been extensively studied for different experimental and disease modalities (DMD, Usher syndrome, hearing loss, Stargardt disease and dysferlinopathy).^92^, ^93^, ^94^, ^95^, ^96^, ^97^, ^98^, ^99^, ^100^, ^101^ Several approaches can be successfully used to assemble large transgene in multiple AAV vectors.

### 5.1 Trans‐splicing and overlapping

In trans‐splicing strategy (TS), the SD (splice donor signal) and SA (splice acceptor signal) are placed at the ends of the split cDNA (Figure 1B) packaged in individual AAV capsid. Upon co‐transfection of cells with dual AAV vectors, the ITRs are concatemerization according to the head‐to‐tail orientation. The SD and SA are located at the ends of cDNA in each AAV vector and are trans‐spliced, followed by the production of full‐sized mRNA and protein.^73^, ^74^, ^75^, ^76^, ^77^, ^78^, ^79^, ^80^, ^81^, ^82^, ^83^, ^84^, ^85^, ^86^, ^87^, ^88^, ^89^, ^90^, ^91^, ^92^, ^93^, ^94^, ^95^, ^96^, ^97^, ^98^, ^99^, ^100^, ^101^, ^102^ The SD and SA sequences for trans‐splicing AAV are vital in promoting the correct trans‐splicing process. The SD and SA sequences should closely adhere to the consensus sequences recognized by the splicing machinery in the cell. For example, the canonical SD sequence typically contains the nucleotide motif ‘AG’ at the 5′ end of the intron, whereas the SA site contains the ‘AG’ dinucleotide at the 3′ end.^103^ Bioinformatic tools are often employed to predict potential SD and acceptor sites within the target genes. Following computational analysis, experimental validation is conducted to confirm the efficacy of the selected splice sequences in promoting trans‐splicing.^104^

Dual‐AAV approach has been recently advanced to UshTher clinical trials aiming to treat retinitis pigmentosa.^92^ However, the results of clinical trials have not been published yet.

An alternative to the TS is a design of overlapping sequences (OV) at the 5′ end of one half of the cDNA and the 3′ end of the second half of the cDNA (Figure 1); thus, the two halves of the cDNA share one OV region, and these overlaps are connected by homologous recombination, which also results in a full‐size gene product.^105^, ^106^, ^107^

The maximum length of OV sequences is limited by the size of the cDNA, and it needs to be experimentally tested for the highest potential region for homologous recombination. The exact size of the OV area remains a matter of debate. For example, Dongsheng Duan, the discoverer of the OV approach, built a functional β‐galactosidase enzyme including a 1 kb overlap.^108^ Nevertheless, shorter (859 nt) overlap was also used to efficiently deliver the 6.2 kb dysferlin‐coding sequence by AAV vector.^107^

Although OV presents several advantages, such as enabling the delivery of larger genes than traditional AAV vectors, there are also limitations associated with this approach. The successful recombination and accurate reassembly of the split gene segments within the target cells may not always be more efficient than is achieved with trans‐splicing vectors, leading to suboptimal or variable expression of the full‐length gene.^109^

### 5.2 Inteins

Intein‐mediated splicing is another method of delivering large genes, which does not fit into an AAV particle. Inteins are genetic elements that can be transcribed and translated and participate in the splicing of exteins (external proteins). Due to the removal of inteins, a site‐specific fusion of its flanking exteins occurs. Inteins are found in many organisms, such as bacteria, fungi, as well as lower plants. The inteins are typically around 200–300 amino acids in length. To accomplish efficient trans‐splicing in the C‐extein, the most important is an amino acid containing a thiol or hydroxyl group (Cys, Ser or Thr) as the first residue.^110^ Overall, inteins are removed through a highly specific and regulated process known as protein splicing, resulting in the production of functional, mature proteins. Splicing with the help of inteins does not require energy consumption, specific proteases or co‐factors.^111^, ^112^ Nevertheless, proper protein folding of both split fragments is a critical prerequisite for efficient protein reconstitution. There are several types of inteins: Full‐sized inteins and mini‐inteins are cis‐splicing inteins, and split‐inteins are trans‐splicing inteins, which means that two subtypes of split‐inteins N‐intein and C‐intein are needed for trans‐splicing, each of which is fused with the opposite end of the extein.^112^, ^113^

In the 2019, Tornabene et al. provided a comparison of the effectiveness of dual AAV vectors and AAV intein‐mediated reconstruction of large proteins, using *EGFP*, *ABCA4* and *CEP290* genes as examples, both in vitro and in vivo.^112^, ^113^, ^114^ In the experiments with HEK293 cells transduction, it was shown that AAV intein‐mediated reconstruction of ABCA4, the accumulation of this protein is higher than with dual AAV vectors, and for CEP290, the expression of the desired protein was observed only with intein‐mediated AAV delivery of the transgene.^114^ It has also been shown in Albino Abca4 ^−^/^−^ and BXD24/TyJ‐Cep290rd16/J mice model that intein‐mediated transgene delivery by AAV is more than twice as efficient than the dual AAV system. Full‐length proteins were detected in 10/11 of AAV‐ABCA4 intein‐injected eyes and 5/10 of AAV‐CEP290 intein‐injected eyes. Conversely, full‐length protein expression was evident in 5/9 and in 0/5 eyes injected with ABCA4 and CEP290 dual AAV vectors, respectively.^114^

Another example of FVIII gene delivery using the inteins and AAV vector was attempted by Esposito et al.^115^ The authors used Npu dnaE split‐inteins and FVIII‐N6 variant with a size of 5 kB for packaging in dual AAV8. The dual AAV8 at the dose of 5 × 10^11^ genome copies was retro‐orbitally injected in C57Bl/6 mice. Dual AAVs effectively expressed the full version of F8‐N6 in the liver cells of mice, thereby achieving the therapeutic level of the FVIII protein.^112^, ^113^, ^114^, ^115^

A hybrid approach using recombination and TS has also been applied for ‘big genes’ transfer with AAV (Figure 1). In this approach, the expression cassette‐coding transgene is broken up and packaged into two independent AAV vectors. A highly recombinogenic DNA sequence introduced at the transgene's termini mediates homologous recombination between split virus genomes in a transgene‐independent manner. In a work by Gosh et al. (2008), a bridging DNA sequence from the human placental alkaline phosphatase (AP) gene accompanied by SD and SA signals was used to deliver B‐galactosidase (LacZ) using AAV.^116^ Minimal recombinogenic sequences of AP (0.26 and 0.27 kb) were sufficient to mediate LacZ reconstitution in M059K cells and in mouse myocytes upon intramuscular injection of 1 × 10^10^ AAV‐6 vg particles/muscle. The data suggest that the hybrid approach showed remarkable transduction efficiency over traditional single trans‐splicing and OV vectors.^117^ Halbert et al. also stated that the split *AP* gene packaged into two AAV‐6 vectors could transduce mouse lung cells as efficiently as did an intact (full‐length) vector.^118^

Trapani et al. compared different strategies of single and dual AAV vectors for the delivery of large ABCA4 and MYO7A genes in vitro and in vivo to mouse and pig retina.^106^ In vitro experiments have shown that all dual AAV vectors proved to be equally effective. However, in vivo results have demonstrated that dual AAV vectors with OV sequences had limited efficiency. The hybrid approach and trans‐splicing are devoid of such problems, as the reconstruction of the complete coding sequence can occur due to ITR‐mediated head‐to‐tail rejoining.^106^, ^116^

In a follow‐up study, the authors have reported the results of hybrid approach efficacy, pharmacokinetics and safety in mice and primates injected with AAV8.MYO7A dual hybrid vector for the treatment of retinitis pigmentosa associated with USH1B. Three doses of AAV8.MYO7A in mice showed MYO7A protein expression between 40% and 67%.^119^

TS requires careful splicing sites design and sequence optimization for effective transgene reassembly into a full‐size transgene. Despite the well‐confirmed feasibility of large gene delivery in basic research, the multiple AAV vectors approach is not favourably accepted for future commercial products. Translation of truncated proteins from non‐trans‐spliced polypeptides and their role in cellular metabolism remain to be thoroughly explored. It has been found that cells transduced by an AAV vector loaded with only one part of a transgene with appropriate genetic regulatory elements can initiate gene expression.^115^ Moreover, the therapeutic efficiency of dual AAV vectors carrying a transgene could correlate with virus load. As was shown by Yan et al. the level of expression of Epo protein delivered by dual AAV2 and TS was higher (9.4‐fold) in the high dosage (4 × 10^11^ vg/muscle) mouse group than in the low (6 × 10^10^ vg/muscle) group at 110 days post treatment.^120^

Inteins operate at the protein level and are considered to facilitate the protein splicing process and full‐length protein synthesis without interfering with the protein structure. However, being of non‐mammalian origin, inteins may hold cryptic immunogenic epitopes and trigger unwanted immune reaction.^121^ This safety concern is particularly relevant if split inteins are combined with Cas‐based systems, as the co‐expression could increase undesirable immune response and eventually compromise gene therapy.^122^, ^123^ Finding a proper and efficient spit site in a transgene might be laborious and time‐consuming work, not always successful. Further, the need to produce multiple AAV vectors for one gene target is associated with higher costs which may hold back the progress of clinical application and drug development.

For the OV and hybrid strategies, the homology sequence is an essential element in determining a split‐gene reconstitution. In the OV strategies, sequences are gene‐specific with only a few variables like overhang length and sequence optimization amenable to modifications. For hybrid approaches, the discovery of better homology sequences could help improve reconstitution levels and efficiency.

Additional aspects of dual AAV efficiency such as viral load, immunogenicity and efficiency of splicing are also critically discussed in the manuscript.

## 6 CIRCULAR PERMUTATION FOR MINIGENES DESIGN

**FIGURE 3:**
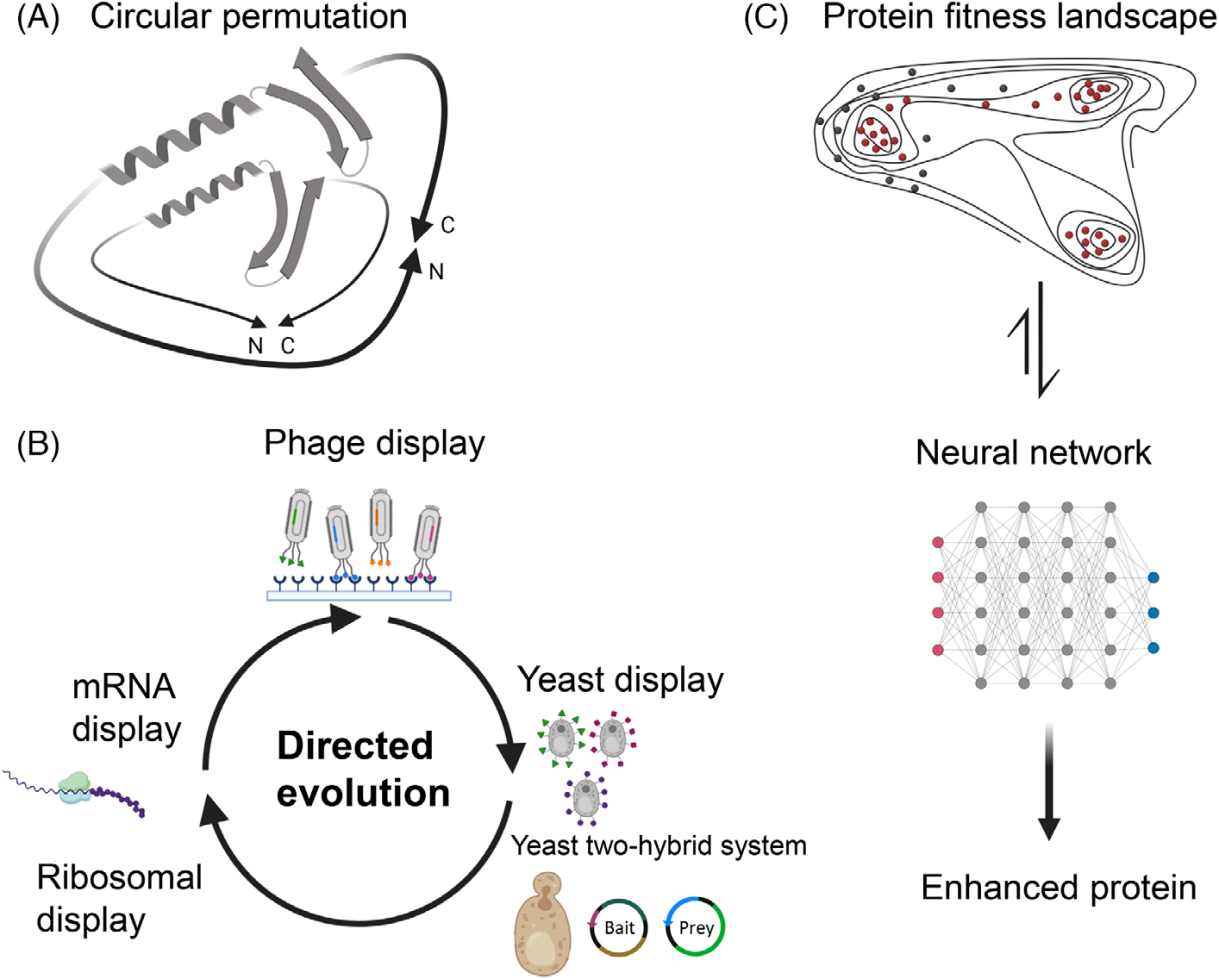
Rational design (A and B) and computational approaches (С) for minimizing protein structures. Circular permutation (A) technique allows screening out non‐essential protein domains and selecting alternative protein structures with desired functionality. The spectrum of rational design tools (B) including library screening with phage, yeast, ribosome and RNA display is a powerful approach to identify protein–protein interaction sites that should be maintained in de novo protein design. Computational approaches for minigene engineering (С) may use fitness landscape experimental data for neural network training to predict de novo protein sequences with enhanced characteristics.

Rational protein design is an extremely targeted and powerful approach, but a laborious and time‐consuming process with sometimes unpredicted outcomes. Traditionally, consecutive or massive parallel (DNA libraries) substitution of amino acids is a method of choice to alter protein functionality or alleviate its function. Circular permutation (CP) is a relatively novel method for protein engineering that has been adapted from nature by scientists. Schematically, CP is a directed evolutionary process of a protein based on the covalent peptide linkage of amino and carboxyl termini of a peptide chain in order to introduce new termini elsewhere in the protein (Figure 3). Structural proximity of the N and C termini of a protein plays a crucial role in efficient permutation.^124^

Circularly permuted proteins often retain conserved three‐dimensional structures and functions. That unique feature may be used for various research and bioengineering applications (e.g. study protein stability, crystallization and de novo protein design).^125^

Circularly permuted proteins have been discovered in nature^126^ and have emerged via gene rearrangements.^127^ Artificial CP has been embraced by genetic engineers to create novel protein sequences with similar 3D structures and some beneficial characteristics and properties.^126^ Taking into account that the amino acid sequence of a protein orchestrates its corrective folding, CP has become a popular technique to discover conformational rearrangements and mobility of the proteins.^128^, ^129^ Fluorescent proteins (i.e. GFP and RFP) are one the most studied objects for CP. The development of a new bright fluorescent permutant has great potential in basic and translational research including biosensors (Ca2+, Zn2+, Cu2+, NH4+ and voltage probes), optogenetics and in vivo imaging.^93^, ^95^, ^96^, ^97^, ^98^, ^99^, ^100^, ^101^, ^102^, ^103^, ^104^, ^105^, ^106^, ^107^, ^108^, ^109^, ^110^, ^111^, ^112^, ^113^, ^114^, ^115^, ^116^, ^117^, ^118^, ^119^, ^120^, ^121^, ^122^, ^123^, ^124^, ^125^, ^126^, ^127^, ^128^, ^129^, ^130^ Examples of circularly permuted proteins are not limited by fluorescent proteins that hold promises in their broad application in protein engineering and biotechnology (phosphoribosyl anthranilate isomerase from yeast,^129^ aspartate transcarbamoylase,^131^ the SH3 domain of α‐spectrin,^132^ chymotrypsin inhibitor,^133^ and thiol/disulfide oxidoreductase DsbA from *Escherichia coli* ^133^, ^134^; and several other proteins). CP is used to reduce proteolytic susceptibility, improve catalytic activity, improve thermostability, identify protein folding dynamics and modify quaternary structure.^124^, ^128^

In our opinion, CP may help to design novel minigens for targeted gene therapy. Performing conformational rearrangements of the sensory domain associated with ligand interaction may create a functional short permutant copy (miniprotein) of a large gene. Circularly permuted protein libraries can be created, and their viability can be subsequently tested by potency tests using relevant cellular models.

Repeated domains arranged in a linear protein molecule (i.e. the Ankyrin repeats, Laminin domains and the Leucine‐rich repeats) also seem to be an appropriate target for the CP application.^135^

Examples from Yao‐Ming Huang et al. demonstrate that several split versions of GFP can be created using CP to reconstitute protein function.^136^ A similar approach might be applied for minigenes/miniprotein design to get insight into the crucial structural elements of a protein. Recent advances in computational biology and bioinformatics may significantly facilitate the analysis and design of circularly permuted proteins. The tools and algorithms for CP are summarized in topic‐related reviews, and we refer the readers to seek the information in Refs. [90, 127, 137, 138] (Figure 2).

Rational protein design can be substantially shaped and boosted by computational approaches. Software‐designed miniproteins with target‐specific binding capacity have been proposed by many groups.^139^, ^140^ Until now, most of them represent nanobinders (antibody‐like structures) to a specific region on a protein surface.^141^, ^142^

The defined crystal structure of a protein of interest and its binding partners is a key component for efficient computational docking of miniproteins and still has a tremendous effect on the rapid design of a minimal functional copy of large genes that do not fit AAV capacity.

Rational design or directed evolution is based on genetic manipulations and sequential in vitro screening of potential candidates with desired functions. Alternatively, machine learning approaches may be used to discover the protein fitness landscape.^143^ New variants of desired protein can be created using preliminary experimental data with subsequent neural network training to predict the functionality of not discovered yet protein variants.^144^ Using GFP, as a model, and machine learning‐driven protein design, Gonzalez Somermeyer et al. discovered that understanding the protein fitness landscape heterogeneity has clear practical application for protein engineering. The combinatorial approach has allowed for a design of a fluorescent protein that differed from parental GFP by 48 mutations. An incredible advantage of machine learning over the evolutionary approach is to speed up the process of protein design and minimize the unpredictability of manually introduced mutations.^144^, ^145^

Another example of the translational application of CP is found in optogenetic engineering. Circularly permutated light‐oxygen‐voltage sensing domain 2 (cpLOV2) from oat phototropin 1 was generated as a potential photoswitchable module for biotechnological research.^146^ Novel cpLOV2 possessed unique caging capabilities and enabled the design of light‐inducible necroptosis via mixed lineage kinase domain‐like protein. Moreover, cpLOV2 was used to construct optoCAR T cells by incorporating photosensory components into an engineered split CD19. Photoinducable activation of primary human CD4^+^ T cells and increased expression of CD69 marker was registered after T cells optoCAR transduction. The study suggests that the cpLOV2 can be used in various clinical and translational settings to effectively modulate T‐cell proliferation, cytokine production and induce tumour cells killing.^146^ It is also worth noting that AAV vectors are compatible with optogenetic modules delivery,^147^ and some of them have already entered clinical trials (NCT02556736, Allergan; NCT03326336, GenSight Biologics).^148^

Although we could not find any direct application of circularly permutated proteins in AAV‐based gene therapeutics yet, we project that AAV vehicles would be an ideal transfer platform to deliver novel designer proteins.

Protein design is a rapidly evolving field indeed and has gained tremendous progress over a few recent years. This review does not go into the latest methods of protein engineering, albeit we would like to highlight their importance for gene therapy RnD. It has been shown that artificial intelligence (i.e. machine learning, deep learning and neural networks) demonstrates great potential in solving biological problems including protein bioengineering.^149^, ^150^ The protein structure prediction algorithm, AlphaFold2, pushed the boundaries of AI‐driven protein design to the next level.^151^ It is worth noting that AI‐based computational tools can craft entirely novel proteins that have never existed in nature. Nevertheless, its biological significance as well as stability and functionality remain a hard task.^152^

We do foresee that machine‐learning approaches will be more frequently used for the determination of a protein function and binding sites based on its structure (Figure 3).

Exciting examples of de novo miniprotein design using (<70 amino acid residues) postulate the feasibility of desirable protein engineering from scratch. Baker's lab demonstrated the accurate prediction and design of small beta‐barrel topologies using the Rosetta energy base‐based method.^153^

There is no doubt that computational power will soon allow us to generate massive miniprotein libraries. Therefore, the bottleneck is production and protein potency testing. Hopefully, available technological platforms for AAV vector production may serve as a way to overcome these hurdles and develop next‐generation therapeutics.

## 7 GENE THERAPY RND LANDSCAPE

**FIGURE 4:**
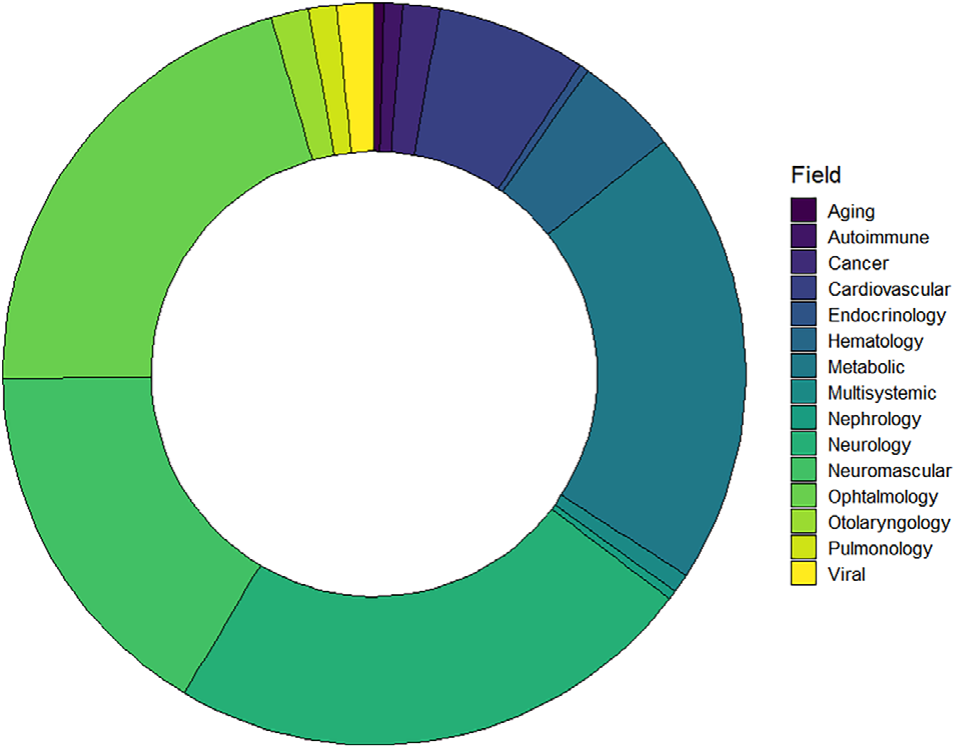
Research and development (RnD) landscape of adeno‐associated virus (AAV)‐based gene therapeutics. AAV vectors are most commonly used for gene therapy of neurological, neuromuscular, metabolic and inherited retinal disorders. The circular diagram shows the number of RnD projects using AAV as a vehicle to deliver therapeutic molecules.

AAV‐based vectors are widely used for basic and translational research to transfer genetic materials into the target cells.^12^ Optogenetics, chemogenetics, genome editing, vaccine developments, immuno‐ and gene therapy are not an exhaustive list of all AAV applications.^154^ Nonetheless, only a few fundamental research studies are translated into commercialized products. A recent meta‐analysis of gene therapy research has beautifully summarized major areas of interest and status of viral‐based therapeutics.^154^ Here, we have attempted to screen and diversify a current gene therapy research landscape with a focus on the biomedical companies’ pipelines (Figure 4). In particular, we were interested in the projects and strategies used to deliver large transgenes with AAV vectors (Table S3). We categorized AAV therapeutics based on the developer, transgene of interest, country, AAV vector type, dose and phase of research for the reader's convenience (Table S1).

**FIGURE 5:**
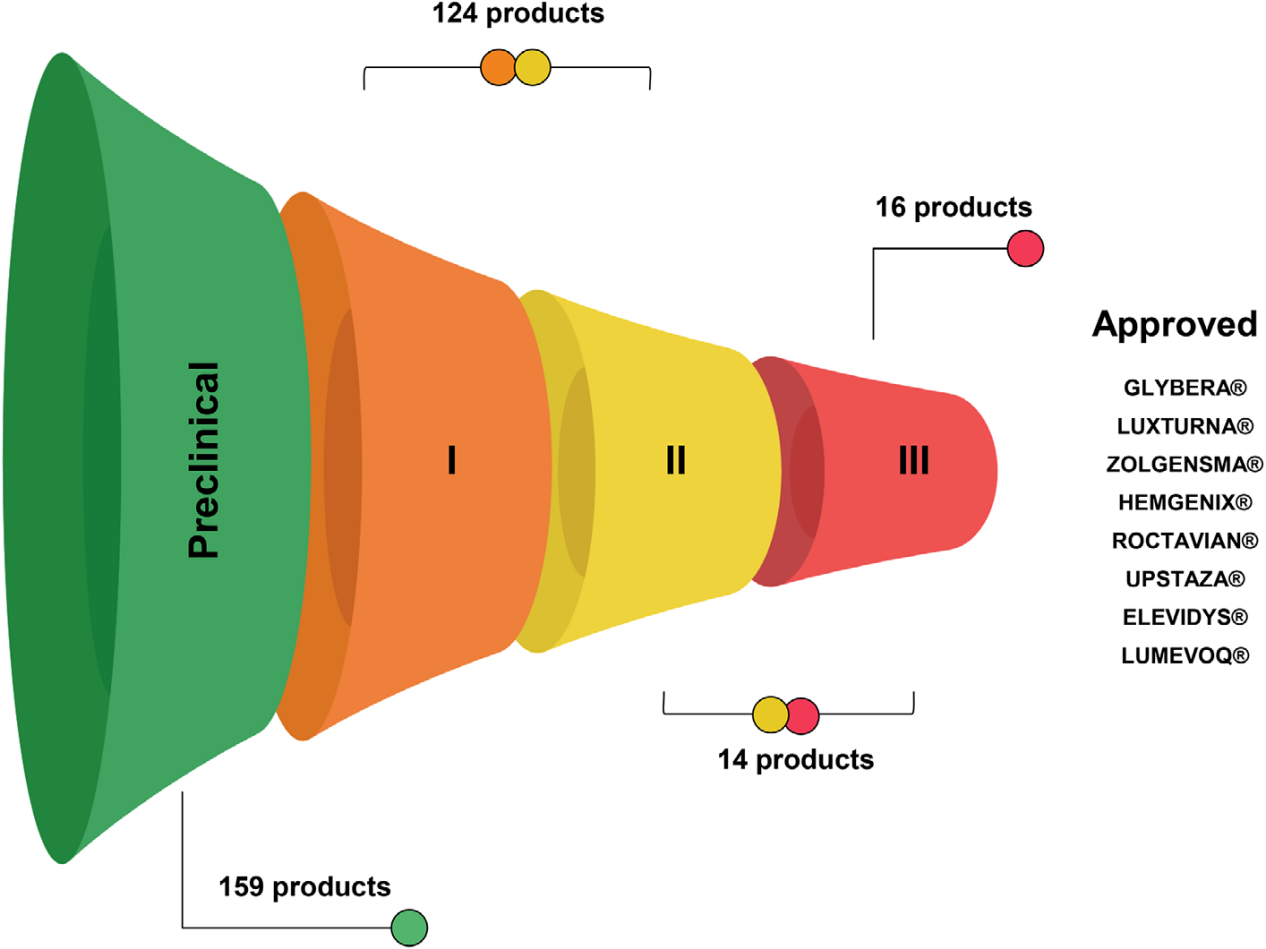
The status and progress of research pipelines from the research and development (RnD) companies using adeno‐associated virus (AAV) as a vector for gene delivery are summarized. The approval rate and intermediate statistics in different clinical phases are presented.

By November 2023, we found 321 AAV vector‐based products designed to treat inherited genetic diseases in various stages of development (Table S1). Among them, 154 products (48%) (Phase I – 27 products (17.5%), Phase I/II – 97 products (63%), Phase I/II/III – 2 products (1.3%), Phase II – 9 products (5.8%), Phase II/III – 3 (1.9%) and Phase III – 16 products (10.4%) have already entered different phases of clinical trials (Figure 5). However, most of the AAV‐based drugs (52%) are still in early stage of development, and preliminary results are not fully disclosed. Only eight products were approved for treatment by the FDA or EMA which is 2.5% of the total number of drugs in development. Despite having a relatively low approval rate, it is important to keep in mind that AAV‐based therapy is a novel approach, and highly probable that the growing number of clinical trials will result in more gene therapeutics in the clinical market soon. It is also worth noting that not all information regarding the vector type, dose, route of administration or transgene structure was available from open sources at the time of this manuscript writing. Several AAV‐based gene therapy clinical trials have been terminated or paused by the developers. Thus, Ultragenyx Pharmaceutical terminated Phase 3 NCT04088734 due to the lack of efficacy of the AAV9 vector carrying *SGSH* gene in patients with advanced mucopolysaccharidosis type III. Another Ultragenyx Pharmaceutical trial (NCT02618915) aimed to treat haemophilia B with AAVrh10 was closed by the sponsor's decision. Similarly, Spark Therapeutics (NCT01620801), FreeLine Therapeutics (NCT05164471), Avigen (NCT00076557), Takeda (NCT04394286) and Sangamo Therapeutics (NCT 02695160) have decided to not further pursue the research to develop AAV‐based therapeutics with coagulation FIX for haemophilia B. According to the results of the aforementioned clinical trials, the reasons for pausing are undisclosed. Business or financial reasons for clinical trial termination are frequently announced by multiple developers regardless of the efficacy of the AAV gene therapy (Table S1).

Overall, a meta‐analysis of 255 clinical trials done in 2022 counted 30 clinical trials on hold, where 18 were due to toxicity.^155^ Resonating and tragic death of four boys in ASPIRO clinical trial aiming to treat patients with X‐linked myotubular myopathy (XLMTM) with AT‐132 (resamirigene bilparvovec) put on hold NCT03199469 initiated by Astellas Pharma. Among the 17 participants who received high dose of the AAV8 vector (3.5 × 10^14^ vg/kg bodyweight), three boys succumbed to fatal liver dysfunction. Among the seven participants injected with a lower dose (1.4 × 10^14^ vg/kg bodyweight), one death was registered.^156^ Despite prior favourable efficacy data for XLMTM gene therapy in murine and canine models, the results of the ASPIRO trial emphasize the importance of understanding the immune mechanisms that may significantly contribute to the safety and efficacy of AAV‐based therapy for individual patients. Moreover, preclinical settings should be taken into consideration for accurate dosing and safe AAV serotype administration.

We anticipate that novel AAV recombinant vectors with induced transduction efficiency or enlarged genome capacity will be discovered and applied. In addition, novel targets for AAV‐based therapy are being announced every week, and the proposed list of AAV therapeutics is continuously expanding. Nevertheless, some gene therapy research has been put on hold despite being accepted for the research portfolio.

Transgene‐wise, the gene therapy landscape is cautiously limited. Thousands of potential gene targets are discovered and suggested for gene therapy, but not all of them seem suitable or attractive for commercialization. We identified 140 genes that were selected by pharmaceutical companies around the world as perspective transgenes. Most of the products (62%) in the research and clinical pipelines are aimed at novel targets to be ‘first‐in‐class’ drugs that offer a new therapeutic approach to treating a disease. The results of any of the research are highly anticipated and extremely demanding.

As was mentioned throughout the review, one of the major hurdles for AAV‐based therapy is the length of a transgene that can fit in the virus capsid. We wondered if there are any approved AAV‐based products or drugs in the research pipelines that address this issue. We summarized all discovered solutions in Table S1.

By the time when the manuscript was written, we found 55 AAV drugs targeting ‘big genes’ (>4.4–4.8 kb) that are currently at different development stages (15 targets are in pipelines, 24 are in clinical trials, and 2 are FDA‐approved) for 38 companies. Intriguingly, the *DMD* gene, despite being one of the biggest genes in the human genome, is a primary target for commercialization chosen by many biotech developers. This fact is partly associated with the results of decades long basic research dedicated to the *DMD* gene structure and functions. Moreover, the recent approval of Elevidys (Sarepta Therapeutics) has triggered additional interest for alternative competitive products. The second popular target for gene addition is an AAV vector carrying a short version of blood FVIII, which was found in nine research pipelines (eight in clinical trials and one is FDA‐approved). Inherited retinal disorders are believed to be a desirable target for gene therapy. We registered that *ABCA*4 minigene is of interest to at least two companies (five projects in the preclinical phase) focusing on Stargardt disease (inherited retinal degeneration). Dysferlinopathy (caused by mutations in the *DYSF* gene) is a focused disease for three projects led by three companies (two in clinical trial phase I/II, one in the preclinical phase). We registered three companies that develop therapy for Wilson's disease with the addition of *ATP7B* gene (two in the clinical phase, one in the preclinical phase). Cystic fibrosis (*CFTRΔR* gene) is a focus of development for the 4D Molecular Therapeutics with two projects in the pipeline.

Autosomal dominant diseases are also a potential target for AAV‐based gene therapy albeit a therapeutic strategy is greatly different. In order to restore, the function of a mutated gene AAV vectors can be adapted to transfer other effector molecules (e.g. siRNA, shRNA or miRNA). It is worth noting that this method can apply to various genetic targets, not just to large genes that go beyond the capacity of the AAV vector. We registered three research projects where AAV vehicles are used to treat Huntington's disease. Huntington's disease is caused by mutations (repetition of the CAG triplet) in the *HTT* gene. Accumulation of toxic mHTT in striatal medium spiny neurons leads to substantial neuronal dysfunction and death occurs in the cerebral cortex. The expression level of malfunctioned mHTT protein can be modulated by either miRNA delivered by AAV‐5 vector (AMT130, UniQure) or siRNA incorporated AAV (VY‐HTT01, Voyager Therapeutics).^157^ Proposed solutions have already entered Phases I and II clinical trials with expected outcomes in 2024 (NCT04120493, NCT04885114). Passage Bio, Inc. has also announced an interest in Huntington disease gene therapy. Detailed information about the methods of delivery and transgene structure is not provided. Astellas has now halted the development and clinical trials of three drugs based on the ASO and AAV for DMD treatment.^158^ The reasons for research termination are not disclosed.

Across the AAV‐based therapeutics in RnD pipelines, we spotted three main approaches that are applied by pharmaceutical companies to circumvent AAV capsid limitations: (i) multiple transduction with two or three AAV vectors; (ii) mini‐version of a functional gene (minigene) that can be packaged in viral capsid and (iii) alternatively, mutations in big genes can be corrected by genome editors or silenced with ASOs or RNA interference.

In this review, we have highlighted several available approaches to target ‘big genes’ for AAV gene addition therapy. The data from the RnD pipelines suggest that transfer of full‐size gene of interest or minimal functional copy is primarily a method of choice for most companies. Delivering a gene that restores the function of full‐length protein and fits into the AAV vector seems a straightforward approach that can bring desired transduction efficiency and minimize vector production costs. We counted on 25 projects that are under development using a miniaturized version of transgenes. MicroDMD is a transgene of interest for 10 projects, B‐domain depleted blood FVIII is used in 5 projects, miniATP7B is used in 3 projects, *CFTR* without regulatory domain is used in 2 projects, and miniABCA4 and miniUSH2A minigenes are announced in individual pipelines (Table S3).

Multiple AAV vectors carrying split transgene can also efficiently deliver large genes. Utilizing cellular trans‐splicing or homologous recombination machinery, fragmented AAV genomes are capable of reassembly followed by gene expression. Nowadays, there are three drugs in research pipelines using this approach for *ABCA4*‐associated Stargardt diseases, dysferlinopathy associated with the *DYSF* gene, Usher syndrome type 2 B caused by mutations in *MYO7A* gene and hearing loss due to *OTOF* malfunction (Table S3). In 2024, promising results of a single‐arm trial involving six children treated with dual‐AAV vectors carrying parts of the *OTOF* gene showed no dose‐limiting toxicity and significant speech perception improvement and partial hearing recovery up to week 26.^159^

Despite being an attractive strategy, some technological aspects may limit the large‐scale production of two or three viral vectors in the same production plant. Efficient delivery of multiple split fragments in target cells by AAV vector also may possess some difficulties in terms of dose calculation and further validation.

## 8 PERSPECTIVES

**FIGURE 6:**
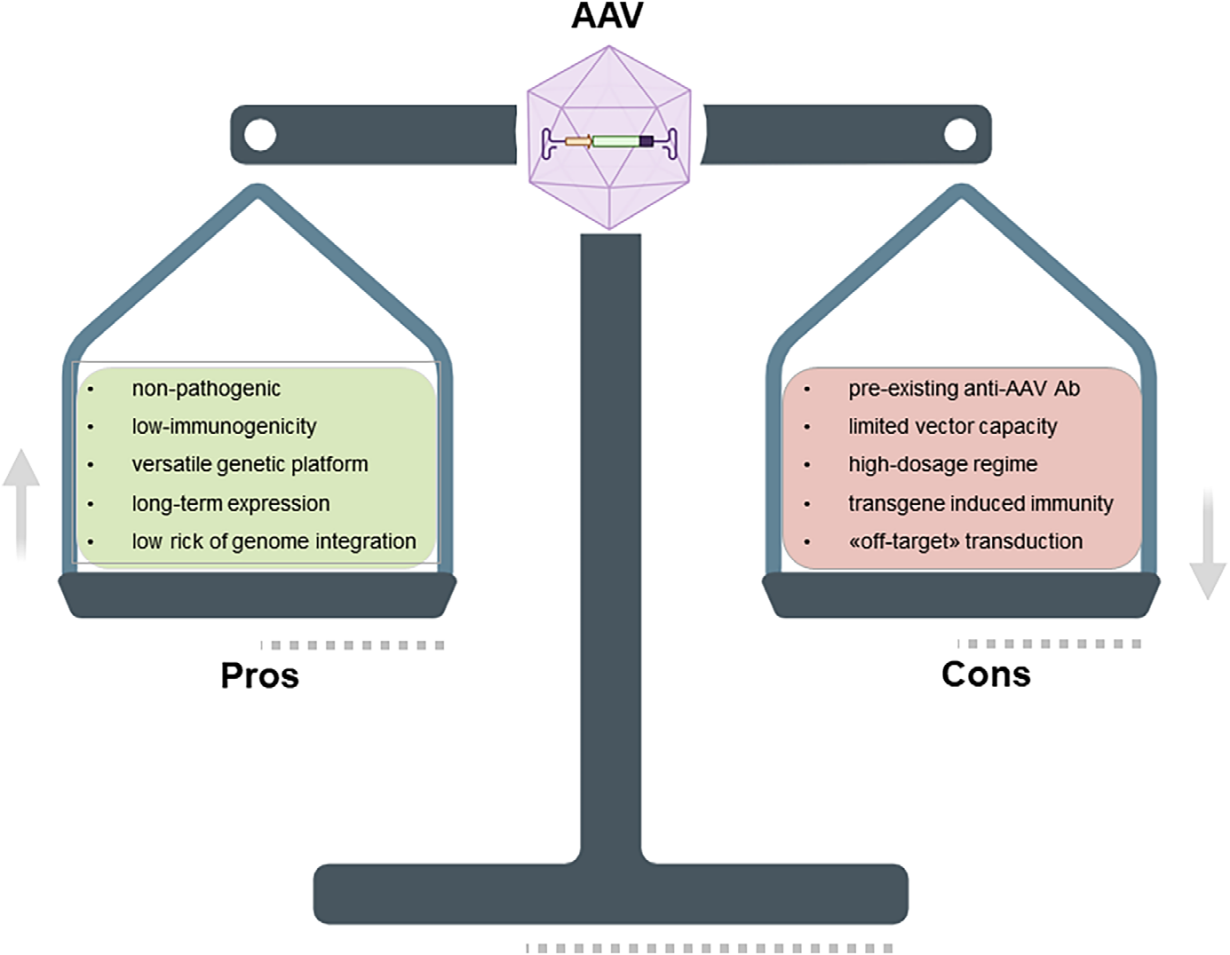
Advantages and limitations of adeno‐associated virus (AAV)‐based gene therapy. Pros and cons are summarized. AAV vectors are one of the most advanced vehicles for translational research. AAV may cargo various genetic materials and molecules and deliver them to the targeted tissue. Nevertheless, several biological and technological limitations should be considered in the research and development (RnD) process of AAV therapeutics.

AAV vectors have been known for decades but their translational potential has been rediscovered relatively recently thanks to the first global approval of *Alipogene tiparvovec* (2012) to treat LPLD, followed by *Voretigene neparvovec*‐rzyl (Luxturna, 2017) for Leber's congenital amaurosis type 2 and *Onasemnogene abeparvovec* (Zolgensma, 2019) to treat SMA.^160^, ^161^ Multiple unique genetic and antigenic characteristics of AAV make it a number one vector of choice for gene delivery: (i) neither wild‐type AAV nor recombinant AAV vectors are pathogenic to humans; (ii) simple and flexible genomic organization allows to generate library of molecular AAV variants; (iii) long‐term transgene expression in dividing and non‐dividing cells; (iv) moderate immunogenicity of viral capsid and (v) large‐scale production and technological platforms are available for massive cGMP‐grade AAV production. A recent systematic review counted more than 200 ongoing clinical trials in gene therapy with AAV vectors as a carrier.^154^ Despite being an attractive and extensively studied viral vector, several obstacles hurdle its clinical applications (Figure 6). In this review, we critically reviewed limited AAV genome capacity (>4.8 kb) and emphasized the approaches that have been developed to address this issue. However, several additional factors should be taken into consideration for the next generation of AAV‐based gene therapy: (i) pre‐existing AAV antibodies, (ii) high‐dosage regimen to achieve high transduction efficiency; (iii) uncontrollable transgene expression; (iv) ‘off‐target’ AAV transduction; (v) transgene‐induced immunity in case of gene addition therapy; (vi) capsid composition and (vii) route of administration. Although we have briefly discussed recent advantages and drawbacks of AAV gene therapy, we refer readers to the most recent topic‐specific articles describing risk factors and limitations of AAV therapy.^162^, ^163^

Clinical durability of therapeutic effect from a transgene delivered by AAV vector is an important characteristic of gene therapy potency. In the best case scenario, a low dose of AAV vector should provide a life‐long expression of a therapeutic gene in a physiological range. In fact, transgene expression supported by AAV genome concatemerization and episomal circulation has been detected for more than 10 years. FDA‐approved *A. tiparvovec* and later *V. neparvovec* demonstrated the durability of response up to 6 and 4 years after drug administration.^164^, ^165^ Excitingly, sustained therapeutic effect after *V. neparvovec* treatment has been recorded after 7.5 years. However, loss of therapeutic response after AAV‐based therapy has also been well documented in animals and humans.^166^, ^167^ It is worth noting that several immunological (innate and adaptive immune response) and non‐immunological (cell turnover in target cells, cellular stress and epigenetic transgene silencing) may drastically affect the therapeutic modality of AAV therapy.^165^, ^168^

To achieve high efficiency of gene delivery to targeted cells by AAV vectors, the dosing regimen should be carefully established. There is no doubt that low doses of AAV cannot provide a sustained level of gene expression and barely may transduce a clinically relevant number of cells to change disease progression. On the other hand, a high dosage regimen of AAV may lead to transduction‐related toxicities.^169^ Moreover, administration of high amounts AAV particles increases the risk of ‘off‐target’ transduction of neighbouring cells causing ‘bystander effects’.^170^, ^171^ In a recent meta‐analysis of AAV clinical applications, Au et al. have reviewed the AAV dosing regimen for targeted (local) and systemic administration.^154^ The lowest dosages for targeted injection were found around 5.8 × 10^9 and 3.5 × 10^13 vg for systemic administration. Despite the growing number of clinical trials with AAV, it is still hard to conclude any reasonable effective therapeutic dose. Targeted administration, when possible, is a preferable way to deliver therapeutic molecules (i.e. transgene, ASO and CRISPR/Cas9) and help to minimize viral dose and mitigate the risk associated with AAV exposure to the immune system and neighbouring cells.

One of the potential clinical limitations of AAV‐based gene therapy is innate and adaptive immune responses to viral elements of a vector. Pre‐existing ААV‐antibodies raise a safety and efficiency concern for gene transfer therapy based on AAV vectors. Recently, three fatal cases among children treated with AAV‐8 expressing the *MTM1* gene for XLMTM were recorded (NCT03199469), and the clinical trial was put on hold till the identification of the cause of death.^172^ Systemic administration of AAV vectors inevitably triggers antiviral innate immune response (e.g. IFN type I and IFN type II) that eventually modulate antibody production by B cells. Neutralizing antibodies (NAbs) can significantly decrease the efficiency of AAV vector transduction and, more importantly, lead to immune pathologies due to immune complex formation.^173^, ^174^ Intriguingly, non‐neutralizing antibodies may promote AAV transduction, but the exact mechanism is not fully discovered yet,^175^ and perhaps, other co‐factors enhance the transduction of AAV vectors, all of these are still subjects of further research.

Several screening studies indicate that anti‐AAV antibodies are frequently detected among the adult population and its serotype prevalence varies in different countries around the world.^176^, ^177^

Nowadays, the identification of total or neutralizing anti‐AAV antibodies is an obligatory prerequisite of efficient and safe gene therapy with AAV vectors.^178^ In case when gene therapy is prescribed but AAV‐serotype‐specific antibodies are detected, several approaches (e.g. plasmapheresis and enzymatic IgG degradation) have been developed to circumvent this limitation.^179^, ^180^ Clinically relevant antibody thresholds (total Ab or only NAbs) and protocols for quantifications should be clearly defined to avoid adverse effects for newly developed AAV‐based therapeutics. Nevertheless, due to the potential risk of pre‐existing anti‐AAV antibodies, seropositive (naive) gene therapy patients have to be informed about associated risks and where possible plasmapheresis should be recommended before vector administration.

We project that the optimization of an AAV dose regimen is a critical step towards mitigation of adverse effects from gene therapy. Gene transfer research should explore the possibilities of delivering a low‐copy (low‐dose) AAV vector to provide a life‐long expression of a therapeutic gene in a physiological range. Artificial intelligence, machine learning and neural network expansion in translation research are inevitable, and we believe that computationally designed transgenes or modified AAV capsids will be proposed soon to help avoid unfavourable immune responses against vectors and facilitate AAV transduction. We foresee that AAV‐based gene therapy will be also more customized and personalized, especially for inherited genetic disorders. Indeed, AAV gene silencing approaches or gene editing with CRISPR‐Cas9 is already patient‐oriented and mutation‐specific. Gene addition technology based on either minigenes or full‐length proteins when applicable may be developed for a broader patient community with a particular pathology.

## AUTHOR CONTRIBUTIONS

Valeria V. Kolesnik wrote, revised manuscript and draw the figures; Ruslan F. Nurtdinov collected and analysed the data; Ezekiel Sola Oloruntimehin wrote, edited and revised the manuscript; Alexander V. Karabelsky wrote discussion and perspectives section; Alexander S. Malogolovkin wrote, edited and revised the manuscript, conceptualized the idea and analysed the data.

## CONFLICT OF INTEREST STATEMENT

The authors declare no conflicts of interest.

## ETHICS STATEMENT

Information from open sources and freely available in the public domain were used in this review.

## Supporting information

Table 1 AAV‐based gene therapeutics.

Table 2 ASO drugs delivered by AAV.

Table 3 ‘Big genes’ targeted by AAV‐based gene therapy.

## Data Availability

The data supporting the findings are available within the article and its supplementary materials.

## References

[1] Pupo A , Fernández A , Low SH , François A , Suárez‐Amarán L , Samulski RJ . AAV vectors: the rubik's cube of human gene therapy. Mol Ther. 2022;30:3515‐3541. 36203359 10.1016/j.ymthe.2022.09.015 PMC9734031

[2] Colella P , Ronzitti G , Mingozzi F . Emerging issues in AAV‐mediated in vivo gene therapy. Mol Ther—Methods Clin Dev. 2018;8:87‐104. 29326962 10.1016/j.omtm.2017.11.007 PMC5758940

[3] Bulcha JT , Wang Yi , Ma H , Tai PWL , Gao G . Viral vector platforms within the gene therapy landscape. Signal Transduction Targeted Ther. 2021;6:1‐24. 10.1038/s41392-021-00487-6 PMC7868676 33558455

[4] Yin H , Kanasty RL , Eltoukhy AA , Vegas AJ , Dorkin JR , Anderson DG . Non‐viral vectors for gene‐based therapy. Nat Rev Genet. 2014;15:541‐555. 25022906 10.1038/nrg3763

[5] Ghosh S , Brown AM , Jenkins C , Campbell K . Viral vector systems for gene therapy: a comprehensive literature review of progress and biosafety challenges. Appl Biosaf. 2020;25:7‐18. 36033383 10.1177/1535676019899502 PMC9134621

[6] De Haan P , Van Diemen FR , Toscano MG . Viral gene delivery vectors: the next generation medicines for immune‐related diseases. Hum Vaccin Immunother. 2021;17:14‐21. 32412865 10.1080/21645515.2020.1757989 PMC7872028

[7] Zu H , Gao D . Non‐viral vectors in gene therapy: recent development, challenges, and prospects. AAPS J. 2021;23:1‐12. 10.1208/s12248-021-00608-7 PMC8171234 34076797

[8] Technological aspects of manufacturing and analytical control of biological nanoparticles. Biotechnol Adv. 2023;64:108122. 36813011 10.1016/j.biotechadv.2023.108122

[9] Kulkarni JA , Witzigmann D , Thomson SB , et al. The current landscape of nucleic acid therapeutics. Nat Nanotechnol. 2021;16:630‐643. 34059811 10.1038/s41565-021-00898-0

[10] Durymanov M , Reineke J . Non‐viral delivery of nucleic acids: insight into mechanisms of overcoming intracellular barriers. Front Pharmacol. 2018;9:398662. 10.3389/fphar.2018.00971 PMC6111240 30186185

[11] Gantenbein B , Tang S , Guerrero J , et al. Non‐viral gene delivery methods for bone and joints. Front Bioeng Biotechnol. 2020;8:598466. 33330428 10.3389/fbioe.2020.598466 PMC7711090

[12] Wang D , Tai PWL , Gao G . Adeno‐associated virus vector as a platform for gene therapy delivery. Nat Rev Drug Discov. 2019;18:358‐378. 30710128 10.1038/s41573-019-0012-9 PMC6927556

[13] Dong J‐Y , Fan P‐D , Frizzell RA . Quantitative analysis of the packaging capacity of recombinant adeno‐associated virus. Hum Gene Ther. 1996;7:2101‐2112. 8934224 10.1089/hum.1996.7.17-2101

[14] Sonntag F , Schmidt K , Kleinschmidt JA . A viral assembly factor promotes AAV2 capsid formation in the nucleolus. Proc Natl Acad Sci U S A. 2010;107:10220‐10225. 20479244 10.1073/pnas.1001673107 PMC2890453

[15] Brister JR , Muzyczka N . Rep‐mediated nicking of the adeno‐associated virus origin requires two biochemical activities, DNA helicase activity and transesterification. J Virol. 1999;73:9325‐9336. 10516041 10.1128/jvi.73.11.9325-9336.1999 PMC112967

[16] Issa SS , Shaimardanova AA , Solovyeva VV , Rizvanov AA . Various AAV serotypes and their applications in gene therapy: an overview. Cells. 2023;12:785. 36899921 10.3390/cells12050785 PMC10000783

[17] Hauck B , Chen L , Xiao W . Generation and characterization of chimeric recombinant AAV vectors. Mol Ther. 2003;7:419‐425. 12668138 10.1016/s1525-0016(03)00012-1 PMC2636682

[18] Landegger LD , Pan B , Askew C , et al. A synthetic AAV vector enables safe and efficient gene transfer to the mammalian inner ear. Nat Biotechnol. 2017;35:280‐284. 28165475 10.1038/nbt.3781 PMC5340646

[19] Samulski RJ , Zhu X , Xiao X , et al. Targeted integration of adeno‐associated virus (AAV) into human chromosome 19. EMBO J. 1991;10:3941‐3950. 1657596 10.1002/j.1460-2075.1991.tb04964.x PMC453134

[20] Duan D . Systemic AAV micro‐dystrophin gene therapy for duchenne muscular dystrophy. Mol Ther. 2018;26:2337‐2356. 30093306 10.1016/j.ymthe.2018.07.011 PMC6171037

[21] Hicks MJ , Rosenberg JB , De BP , et al. AAV‐directed persistent expression of a gene encoding anti‐nicotine antibody for smoking cessation. Sci Transl Med. 2012;4:140ra87. 10.1126/scitranslmed.3003611 PMC3622954 22745437

[22] Manno CS , Chew AJ , Hutchison S , et al. AAV‐mediated factor IX gene transfer to skeletal muscle in patients with severe hemophilia B. Blood. 2003;101:2963‐2972. 12515715 10.1182/blood-2002-10-3296

[23] Termini JM , Martinez‐Navio JM , Gao G , Fuchs SP , Desrosiers RC . Glycoengineering of AAV‐delivered monoclonal antibodies yields increased ADCC activity. Mol. Ther—Methods Clin Dev. 2021;20:204‐217. 33426147 10.1016/j.omtm.2020.11.001 PMC7782200

[24] Gene therapy clinical trials, where do we go? An overview. Biomed Pharmacother. 2022;153:113324. 35779421 10.1016/j.biopha.2022.113324

[25] Yang Q , Tang Y , Imbrogno K , et al. AAV‐based shRNA silencing of NF‐κB ameliorates muscle pathologies in mdx mice. Gene Ther. 2012;19:1196‐1204. 22278411 10.1038/gt.2011.207

[26] Tomar RS , Matta H , Chaudhary PM . Use of adeno‐associated viral vector for delivery of small interfering RNA. Oncogene. 2003;22:5712‐5715. 12944921 10.1038/sj.onc.1206733

[27] Wang D , Zhang F , Gao G . CRISPR‐based therapeutic genome editing: strategies and in vivo delivery by AAV vectors. Cell. 2020;181:136‐150. 32243786 10.1016/j.cell.2020.03.023 PMC7236621

[28] Davis JR , Wang X , Witte IP , et al. Efficient in vivo base editing via single adeno‐associated viruses with size‐optimized genomes encoding compact adenine base editors. Nat Biomed Eng. 2022;6:1272‐1283. 35902773 10.1038/s41551-022-00911-4 PMC9652153

[29] Aslesh T , Yokota T . Restoring SMN expression: an overview of the therapeutic developments for the treatment of spinal muscular atrophy. Cells. 2022;11:417. 35159227 10.3390/cells11030417 PMC8834523

[30] Peccate C , Mollard A , Le Hir M , et al. Antisense pre‐treatment increases gene therapy efficacy in dystrophic muscles. Hum Mol Genet. 2016;25:3555‐3563. 27378686 10.1093/hmg/ddw201

[31] https://www.fda.gov/news‐events/press‐announcements/fda‐approves‐first‐gene‐therapy‐adults‐severe‐hemophilia

[32] European Medicines Agency . First gene therapy to treat haemophilia B. European Medicines Agency; 2022. https://www.ema.europa.eu/en/news/first‐gene‐therapy‐treat‐haemophilia‐b

[33] Center for Biologics Evaluation & Research . Approved cellular and gene therapy products. U.S. Food and Drug Administration; 2023. https://www.fda.gov/vaccines‐blood‐biologics/cellular‐gene‐therapy‐products/approved‐cellular‐and‐gene‐therapy‐products

[34] Allocca M , Doria M , Petrillo M , et al. Serotype‐dependent packaging of large genes in adeno‐associated viral vectors results in effective gene delivery in mice. J Clin Invest. 2008;118:1955‐1964. 18414684 10.1172/JCI34316 PMC2298836

[35] Wu Z , Yang H , Colosi P . Effect of genome size on AAV vector packaging. Mol Ther. 2010;18:80‐86. 19904234 10.1038/mt.2009.255 PMC2839202

[36] Dong B , Nakai H , Xiao W . Characterization of genome integrity for oversized recombinant AAV vector. Mol Ther. 2010;18:87‐92. 19904236 10.1038/mt.2009.258 PMC2803017

[37] Shao L , Shen W , Wang S , Qiu J . Recent advances in molecular biology of human bocavirus 1 and its applications. Front Microbiol. 2021;12:696604. 34220786 10.3389/fmicb.2021.696604 PMC8242256

[38] Qiu J , Söderlund‐Venermo M , Young NS . Human parvoviruses. Clin Microbiol Rev. 2017;30:43‐113. 27806994 10.1128/CMR.00040-16 PMC5217800

[39] Molecular dynamics simulation for rational protein engineering: present and future prospectus. J Mol Graph Model. 2018;84:43‐53. 29909273 10.1016/j.jmgm.2018.06.009

[40] Tertrais M , Bouleau Y , Emptoz A , et al. Viral transfer of mini‐otoferlins partially restores the fast component of exocytosis and uncovers ultrafast endocytosis in auditory hair cells of otoferlin knock‐out mice. J Neurosci. 2019;39:3394‐3411. 30833506 10.1523/JNEUROSCI.1550-18.2018 PMC6495124

[41] Ivanchenko MV , Hathaway DM , Klein AJ , et al. Mini‐PCDH15 gene therapy rescues hearing in a mouse model of usher syndrome type 1F. Nat Commun. 2023;14:2400. 37100771 10.1038/s41467-023-38038-y PMC10133396

[42] Wang W , Vandenberghe LH . Miniaturization of usher syndrome type 2A gene for AAV mediated gene therapy. Invest Ophthalmol Vis Sci. 2021;62:1194‐1194.

[43] Hoffman EP , Brown RH , Kunkel LM . Dystrophin: the protein product of the Duchenne muscular dystrophy locus. Cell. 1987;51:919‐928. 3319190 10.1016/0092-8674(87)90579-4

[44] Rando TA . The dystrophin‐glycoprotein complex, cellular signaling, and the regulation of cell survival in the muscular dystrophies. Muscle Nerve. 2001;24:1575‐1594. 11745966 10.1002/mus.1192

[45] Van Deutekom JCT , Van Ommen G‐JB . Advances in Duchenne muscular dystrophy gene therapy. Nat Rev Genet. 2003;4:774‐783. 14526374 10.1038/nrg1180

[46] Birch SM , Lawlor MW , Conlon TJ , et al. Assessment of systemic AAV‐microdystrophin gene therapy in the GRMD model of Duchenne muscular dystrophy. Sci Transl Med. 2023;15:eabo1815. 36599002 10.1126/scitranslmed.abo1815 PMC11107748

[47] Manini A , Abati E , Nuredini A , Corti S , Comi GP . Adeno‐associated virus (AAV)‐mediated gene therapy for Duchenne muscular dystrophy: the issue of transgene persistence. Front Neurol. 2021;12:814174. 35095747 10.3389/fneur.2021.814174 PMC8797140

[48] Lenting PJ , Van Mourik JA , Mertens K . The life cycle of coagulation factor VIII in view of its structure and function. Blood. 1998;92:3983‐3996. 9834200

[49] Hu Z , Li Z , Wu Y , et al. Targeted B‐domain deletion restores F8 function in human endothelial cells and mice. Signal Transduction Targeted Ther. 2022;7:1‐3. 10.1038/s41392-022-01016-9 PMC9207027 35718817

[50] Domenger C , Grimm D . Next‐generation AAV vectors‐do not judge a virus (only) by its cover. Hum Mol Genet. 2019;28:R3‐R14. 31261383 10.1093/hmg/ddz148

[51] Li C , Samulski RJ . Engineering adeno‐associated virus vectors for gene therapy. Nat Rev Genet. 2020;21:255‐272. 32042148 10.1038/s41576-019-0205-4

[52] Suoranta T , Laham‐Karam N , Ylä‐Herttuala S . Strategies to improve safety profile of AAV vectors. Front Mol Med. 2022;2:1054069. 10.3389/fmmed.2022.1054069 PMC11285686 39086961

[53] Becker J , Fakhiri J , Grimm D . Fantastic AAV gene therapy vectors and how to find them‐random diversification, rational design and machine learning. Pathogens. 2022;11:756. 35890005 10.3390/pathogens11070756 PMC9318892

[54] Nieuwenhuis B , Haenzi B , Hilton S , et al. Optimization of adeno‐associated viral vector‐mediated transduction of the corticospinal tract: comparison of four promoters. Gene Ther. 2021;28:56‐74. 32576975 10.1038/s41434-020-0169-1 PMC7902269

[55] Nieuwenhuis B , Laperrousaz E , Tribble JR , et al. Improving adeno‐associated viral (AAV) vector‐mediated transgene expression in retinal ganglion cells: comparison of five promoters. Gene Ther. 2023;30:503‐519. 36635457 10.1038/s41434-022-00380-z PMC10284706

[56] Skopenkova VV , Egorova TV , Bardina MV . Muscle‐specific promoters for gene therapy. Acta Naturae. 2021;13:47‐58. 10.32607/actanaturae.11063 PMC8084301 33959386

[57] Soroka AB , Feoktistova SG , Mityaeva ON , Volchkov PY . Gene therapy approaches for the treatment of hemophilia B. Int J Mol Sci. 2023;24:10766. 37445943 10.3390/ijms241310766 PMC10341900

[58] ROCTAVIANTM (valoctocogene roxaparvovec‐rvox). BioMarin https://www.biomarin.com/our‐treatments/products/roctavian‐valoctocogene‐roxaparvovec‐rvox/(2023)

[59] Chai S , Wakefield L , Norgard M , et al. Strong ubiquitous micro‐promoters for recombinant adeno‐associated viral vectors. Mol Ther Methods Clin Dev. 2023;29:504‐512. 37287749 10.1016/j.omtm.2023.05.013 PMC10241652

[60] Yan Z , Zak R , Zhang Y , Engelhardt JF . Inverted terminal repeat sequences are important for intermolecular recombination and circularization of adeno‐associated virus genomes. J Virol. 2005;79:364‐379. 15596830 10.1128/JVI.79.1.364-379.2005 PMC538689

[61] Earley LF , Conatser LM , Lue VM , et al. Adeno‐associated virus serotype‐specific inverted terminal repeat sequence role in vector transgene expression. Hum Gene Ther. 2020;31:151‐162. 31914802 10.1089/hum.2019.274 PMC7047122

[62] Duan D , Sharma P , Yang J , et al. Circular intermediates of recombinant adeno‐associated virus have defined structural characteristics responsible for long‐term episomal persistence in muscle tissue. J Virol. 1999;73:861‐861. 10.1128/jvi.72.11.8568-8577.1998 PMC110267 9765395

[63] Mccarty DM , Fu H , Monahan PE , Toulson CE , Naik P , Samulski RJ . Adeno‐associated virus terminal repeat (TR) mutant generates self‐complementary vectors to overcome the rate‐limiting step to transduction in vivo. Gene Ther. 2003;10:2112‐2118. 14625565 10.1038/sj.gt.3302134

[64] Mccarty Dm , Monahan Pe , Samulski Rj . Self‐complementary recombinant adeno‐associated virus (scAAV) vectors promote efficient transduction independently of DNA synthesis. Gene Ther. 2001;8:1248‐1254. 11509958 10.1038/sj.gt.3301514

[65] Wu J , Zhao W , Zhong Li , et al. Self‐complementary recombinant adeno‐associated viral vectors: packaging capacity and the role of rep proteins in vector purity. Hum Gene Ther. 2007;18:171‐182. 17328683 10.1089/hum.2006.088

[66] Mccarty DM . Self‐complementary AAV vectors; advances and applications. Mol Ther. 2008;16:1648‐1656. 18682697 10.1038/mt.2008.171

[67] Savy A , Dickx Y , Nauwynck L , Bonnin D , Merten O‐W , Galibert L . Impact of inverted terminal repeat integrity on rAAV8 production using the baculovirus/Sf9 cells system. Hum Gene Ther Methods. 2017;28:277‐289. 28967288 10.1089/hgtb.2016.133 PMC5655423

[68] Ryan JH , Zolotukhin S , Muzyczka N . Sequence requirements for binding of Rep68 to the adeno‐associated virus terminal repeats. J Virol. 1996;70:1542‐1553. 8627673 10.1128/jvi.70.3.1542-1553.1996 PMC189976

[69] Shitik EM , Shalik IK , Yudkin DV . AAV‐ based vector improvements unrelated to capsid protein modification. Front Med. 2023;10:1106085. 10.3389/fmed.2023.1106085 PMC9935841 36817775

[70] Liu Y , Joo K‐I , Wang P . Endocytic processing of adeno‐associated virus type 8 vectors for transduction of target cells. Gene Ther. 2013;20:308‐317. 22622241 10.1038/gt.2012.41

[71] Hanlon KS , Meltzer JC , Buzhdygan T , et al. Selection of an efficient AAV vector for robust CNS transgene expression. Mol Ther Methods Clin Dev. 2019;15:320‐332. 31788496 10.1016/j.omtm.2019.10.007 PMC6881693

[72] Choi J‐H , Yu N‐K , Baek Gi‐C , et al. Optimization of AAV expression cassettes to improve packaging capacity and transgene expression in neurons. Mol Brain. 2014;7:17. 24618276 10.1186/1756-6606-7-17 PMC3975461

[73] Fuentes CM , Schaffer DV . Adeno‐associated virus‐mediated delivery of CRISPR‐Cas9 for genome editing in the central nervous system. Curr Opin Biomed Eng. 2018;7:33‐41. 34046535 10.1016/j.cobme.2018.08.003 PMC8153090

[74] Kim DoY , Lee JMi , Moon SuB , et al. Efficient CRISPR editing with a hypercompact Cas12f1 and engineered guide RNAs delivered by adeno‐associated virus. Nat Biotechnol. 2022;40:94‐102. 34475560 10.1038/s41587-021-01009-z PMC8763643

[75] Dominski Z , Kole R . Restoration of correct splicing in thalassemic pre‐mRNA by antisense oligonucleotides. Proc Natl Acad Sci U S A. 1993;90:8673‐8677. 8378346 10.1073/pnas.90.18.8673 PMC47420

[76] Abaji M , Gorokhova S , Da Silva N , et al. Novel exon‐skipping therapeutic approach for the DMD gene based on asymptomatic deletions of exon 49. Genes. 2022;13:1277. 35886062 10.3390/genes13071277 PMC9323532

[77] Schellens RTW , Broekman S , Peters T , et al. A protein domain‐oriented approach to expand the opportunities of therapeutic exon skipping for ‐associated retinitis pigmentosa. Mol Ther Nucleic Acids. 2023;32:980‐994. 37313440 10.1016/j.omtn.2023.05.020 PMC10258241

[78] Leier A , Moore M , Liu H , et al. Targeted exon skipping of exon 17 as a therapeutic for neurofibromatosis type I. Mol Ther Nucleic Acids. 2022;28:261‐278. 35433111 10.1016/j.omtn.2022.03.011 PMC8983316

[79] Tabebordbar M , Zhu K , Cheng JKW , et al. In vivo gene editing in dystrophic mouse muscle and muscle stem cells. Science. 2016;351:407‐411. 26721686 10.1126/science.aad5177 PMC4924477

[80] Ran FA , Cong Le , Yan WX , et al. In vivo genome editing using *Staphylococcus aureus* Cas9. Nature. 2015;520:186‐191. 25830891 10.1038/nature14299 PMC4393360

[81] Long C , Amoasii L , Mireault AA , et al. Postnatal genome editing partially restores dystrophin expression in a mouse model of muscular dystrophy. Science. 2016;351:400‐403. 26721683 10.1126/science.aad5725 PMC4760628

[82] Yang Y , Wang L , Bell P , et al. A dual AAV system enables the Cas9‐mediated correction of a metabolic liver disease in newborn mice. Nat Biotechnol. 2016;34:334‐338. 26829317 10.1038/nbt.3469 PMC4786489

[83] Behr M , Zhou J , Xu B , Zhang H . In vivo delivery of CRISPR‐Cas9 therapeutics: progress and challenges. Acta Pharm Sin B. 2021;11:2150‐2171. 34522582 10.1016/j.apsb.2021.05.020 PMC8424283

[84] Yip B . Recent Advances in CRISPR/Cas9 Delivery Strategies. Biomolecules. 2020;10:839. 32486234 10.3390/biom10060839 PMC7356196

[85] Marino M , Holt MG . AAV vector‐mediated antibody delivery (A‐MAD) in the central nervous system. Front Neurol. 2022;13:870799. 35493843 10.3389/fneur.2022.870799 PMC9039256

[86] Maeder ML , Stefanidakis M , Wilson CJ , et al. Development of a gene‐editing approach to restore vision loss in leber congenital amaurosis type 10. Nat Med. 2019;25:229‐233. 30664785 10.1038/s41591-018-0327-9

[87] LeMieux J . Editas medicine pauses trial of CRISPR‐Cas9 treatment for blindness disorder. GEN—Genetic Engineering and Biotechnology News https://www.genengnews.com/news/editas‐medicine‐pauses‐trial‐of‐crispr‐cas9‐treatment‐for‐blindness‐disorder/(2022)

[88] Schümperli D , Pillai RS . The special Sm core structure of the U7 snRNP: far‐reaching significance of a small nuclear ribonucleoprotein. Cell Mol Life Sci. 2004;61:2560‐2570. 15526162 10.1007/s00018-004-4190-0 PMC11924553

[89] Wein N , Vetter TA , Vulin A , et al. Systemic delivery of an AAV9 exon‐skipping vector significantly improves or prevents features of Duchenne muscular dystrophy in the Dup2 mouse. Mol Ther Methods Clin Dev. 2022;26:279‐293. 35949298 10.1016/j.omtm.2022.07.005 PMC9356240

[90] Aupy P , Zarrouki F , Sandro Q , et al. Long‐term efficacy of AAV9‐U7snRNA‐mediated exon 51 skipping in mdx52 mice. Mol Ther Methods Clin Dev. 2020;17:1037‐1047. 32462052 10.1016/j.omtm.2020.04.025 PMC7240049

[91] Duan D , Sharma P , Yang J , et al. Circular intermediates of recombinant adeno‐associated virus have defined structural characteristics responsible for long‐term episomal persistence in muscle tissue. J Virol. 1998;72:8568‐8577. 9765395 10.1128/jvi.72.11.8568-8577.1998 PMC110267

[92] Clinical trial of gene therapy with dual AAV vectors for retinitis pigmentosa in patients with Usher syndrome type IB. CORDIS|European Commission; 2022. https://cordis.europa.eu/project/id/754848 doi:10.3030/754848

[93] Kodippili K , Hakim CH , Pan X , et al. Dual AAV gene therapy for Duchenne muscular dystrophy with a 7‐kb mini‐dystrophin gene in the canine model. Hum Gene Ther. 2018;29:299‐311. 28793798 10.1089/hum.2017.095 PMC5865264

[94] Pickar‐Oliver A , Gough V , Bohning JD , et al. Full‐length dystrophin restoration via targeted exon integration by AAV‐CRISPR in a humanized mouse model of Duchenne muscular dystrophy. Mol Ther. 2021;29:3243‐3257. 34509668 10.1016/j.ymthe.2021.09.003 PMC8571168

[95] Riaz S , Sethna S , Duncan T , et al. Dual AAV‐based PCDH15 gene therapy achieves sustained rescue of visual function in a mouse model of usher syndrome 1F. Mol Ther. 2023;31:3490–3501. doi:10.1016/j.ymthe.2023.10.017 37864333 PMC10727994

[96] Ivanchenko MV , Hathaway DM , Mulhall EM , et al. PCDH15 dual‐AAV gene therapy for deafness and blindness in usher syndrome type 1F. Biorxiv. 2023. doi:10.1101/2023.11.09.566447 PMC11601915 39441757

[97] Al‐Moyed H , Cepeda AP , Jung S , Moser T , Kügler S , Reisinger E . A dual‐AAV approach restores fast exocytosis and partially rescues auditory function in deaf otoferlin knock‐out mice. EMBO Mol Med. 2019;11. 10.15252/emmm.201809396 PMC6328916 30509897

[98] Al‐Moyed H , Cepeda AP , Jung S , Moser T , Kügler S , Reisinger E . A dual‐AAV approach restores fast exocytosis and partially rescues auditory function in deaf otoferlin knock‐out mice. EMBO Mol Med. 2019;11:e9396. 30509897 10.15252/emmm.201809396 PMC6328916

[99] Lostal W , Bartoli M , Bourg N , et al. Efficient recovery of dysferlin deficiency by dual adeno‐associated vector‐mediated gene transfer. Hum Mol Genet. 2010;19:1897‐1907. 20154340 10.1093/hmg/ddq065

[100] Potter RA , Griffin DA , Sondergaard PC , et al. Systemic delivery of dysferlin overlap vectors provides long‐term gene expression and functional improvement for dysferlinopathy. Hum Gene Ther. 2018;29:749‐762. 28707952 10.1089/hum.2017.062 PMC6066196

[101] Sondergaard PC , Griffin DA , Pozsgai ER , et al. AAV.Dysferlin overlap vectors restore function in dysferlinopathy animal models. Ann Clin Transl Neurol. 2015;2:256‐270. 25815352 10.1002/acn3.172 PMC4369275

[102] Lai Yi , Yue Y , Liu M , et al. Efficient in vivo gene expression by trans‐splicing adeno‐associated viral vectors. Nat Biotechnol. 2005;23:1435‐1439. 16244658 10.1038/nbt1153 PMC2581721

[103] Stutika C , Gogol‐Döring A , Botschen L , et al. A comprehensive RNA sequencing analysis of the adeno‐associated virus (AAV) type 2 transcriptome reveals novel AAV transcripts, splice variants, and derived proteins. J Virol. 2016;90:1278‐1289. 26559843 10.1128/JVI.02750-15 PMC4719636

[104] Farris KD , Pintel DJ . Improved splicing of adeno‐associated viral (AAV) capsid protein‐supplying pre‐mRNAs leads to increased recombinant AAV vector production. Hum Gene Ther. 2008;19:1421‐1427. 18785816 10.1089/hum.2008.118 PMC2940631

[105] McClements ME , MacLaren RE . Adeno‐associated virus (AAV) dual vector strategies for gene therapy encoding large transgenes. Yale J Biol Med. 2017;90:611‐623. 29259525 PMC5733846

[106] Trapani I , Colella P , Sommella A , et al. Effective delivery of large genes to the retina by dual AAV vectors. EMBO Mol Med. 2014;6:194‐211. 24150896 10.1002/emmm.201302948 PMC3927955

[107] Pryadkina M , Lostal W , Bourg N , et al. A comparison of AAV strategies distinguishes overlapping vectors for efficient systemic delivery of the 6.2 kb dysferlin coding sequence. Mol Ther—Methods Clin Dev. 2015;2:15009. 26029720 10.1038/mtm.2015.9 PMC4445010

[108] Duan D , Yue Y , Engelhardt JF . Expanding AAV packaging capacity with trans‐splicing or overlapping vectors: a quantitative comparison. Mol Ther. 2001;4:383‐391. 11592843 10.1006/mthe.2001.0456

[109] Reisinger E . Dual‐AAV delivery of large gene sequences to the inner ear. Hear Res. 2020;394:107857. 31810595 10.1016/j.heares.2019.107857

[110] Shah NH , Eryilmaz E , Cowburn D , Muir TW . Extein residues play an intimate role in the rate‐limiting step of protein trans‐splicing. J Am Chem Soc. 2013;135:5839‐5847. 23506399 10.1021/ja401015p PMC3630739

[111] Novikova O , Topilina N , Belfort M . Enigmatic distribution, evolution, and function of inteins. J Biol Chem. 2014;289:14490‐14497. 24695741 10.1074/jbc.R114.548255 PMC4031506

[112] Mills KV , Johnson MA , Perler FB . Protein splicing: how inteins escape from precursor proteins. J Biol Chem. 2014;289:14498‐14505. 24695729 10.1074/jbc.R113.540310 PMC4031507

[113] Stevens AJ , Brown ZZ , Shah NH , Sekar G , Cowburn D , Muir TW . Design of a split intein with exceptional protein splicing activity. J Am Chem Soc. 2016;138:2162‐2165. 26854538 10.1021/jacs.5b13528 PMC4894280

[114] Tornabene P , Trapani I , Minopoli R , et al. Intein‐mediated protein trans‐splicing expands adeno‐associated virus transfer capacity in the retina. Sci Transl Med. 2019;11:eaav4523. 31092694 10.1126/scitranslmed.aav4523 PMC6863751

[115] Esposito F , Lyubenova H , Tornabene P , et al. Liver gene therapy with intein‐mediated F8 trans‐splicing corrects mouse haemophilia A. EMBO Mol Med. 2022;14:e15199. 35491676 10.15252/emmm.202115199 PMC9174883

[116] Ghosh A , Yue Y , Lai Yi , Duan D . A hybrid vector system expands adeno‐associated viral vector packaging capacity in a transgene‐independent manner. Mol Ther. 2008;16:124‐130. 17984978 10.1038/sj.mt.6300322

[117] Ghosh A , Yue Y , Duan D . Efficient transgene reconstitution with hybrid dual AAV vectors carrying the minimized bridging sequences. Hum Gene Ther. 2011;22:77‐83. 20662564 10.1089/hum.2010.122 PMC3025179

[118] Halbert CL , Allen JM , Miller AD . Efficient mouse airway transduction following recombination between AAV vectors carrying parts of a larger gene. Nat Biotechnol. 2002;20:697‐701. 12089554 10.1038/nbt0702-697

[119] Ferla R , Dell'aquila F , Doria M , et al. Efficacy, pharmacokinetics, and safety in the mouse and primate retina of dual AAV vectors for usher syndrome type 1B. Mol Ther Methods Clin Dev. 2023;28:396‐411. 36910588 10.1016/j.omtm.2023.02.002 PMC9996380

[120] Yan Z , Zhang Y , Duan D , Engelhardt JF . Trans‐splicing vectors expand the utility of adeno‐associated virus for gene therapy. Proc Natl Acad Sci U S A. 2000;97:6716‐6721. 10841568 10.1073/pnas.97.12.6716 PMC18714

[121] Tharappel AM , Li Z , Li H . Inteins as drug targets and therapeutic tools. Front Mol Biosci. 2022;9:821146. 35211511 10.3389/fmolb.2022.821146 PMC8861304

[122] Riedmayr LM , Hinrichsmeyer KS , Thalhammer SB , et al. mRNA trans‐splicing dual AAV vectors for (epi)genome editing and gene therapy. Nat Commun. 2023;14:6578. 37852949 10.1038/s41467-023-42386-0 PMC10584818

[123] Hakim CH , Kumar SRP , Pérez‐López DO , et al. Cas9‐specific immune responses compromise local and systemic AAV CRISPR therapy in multiple dystrophic canine models. Nat Commun. 2021;12:6769. 34819506 10.1038/s41467-021-26830-7 PMC8613397

[124] Bliven S , Prlić A . Circular permutation in proteins. PLoS Comput Biol. 2012;8:e1002445. 22496628 10.1371/journal.pcbi.1002445 PMC3320104

[125] Schwartz TU , Walczak R , Blobel G . Circular permutation as a tool to reduce surface entropy triggers crystallization of the signal recognition particle receptor beta subunit. Protein Sci. 2004;13:2814‐2818. 15340174 10.1110/ps.04917504 PMC2286555

[126] Uliel S , Fliess A , Unger R . Naturally occurring circular permutations in proteins. Protein Eng. 2001;14:533‐542. 11579221 10.1093/protein/14.8.533

[127] Yu Y , Lutz S . Circular permutation: a different way to engineer enzyme structure and function. Trends Biotechnol. 2011;29:18‐25. 21087800 10.1016/j.tibtech.2010.10.004

[128] Kostyuk AI , Demidovich AD , Kotova DA , Belousov VV , Bilan DS . Circularly permuted fluorescent protein‐based indicators: history, principles, and classification. Int J Mol Sci. 2019;20:4200. 31461959 10.3390/ijms20174200 PMC6747460

[129] Luger K , Hommel U , Herold M , Hofsteenge J , Kirschner K . Correct folding of circularly permuted variants of a beta alpha barrel enzyme in vivo. Science. 1989;243:206‐210. 2643160 10.1126/science.2643160

[130] Truong J , Hsieh Yu‐F , Truong L , Jia G , Hammond MC . Designing fluorescent biosensors using circular permutations of riboswitches. Methods. 2018;143:102‐109. 29458090 10.1016/j.ymeth.2018.02.014 PMC6051913

[131] Zhang P , Schachman HK . In vivo formation of allosteric aspartate transcarbamoylase containing circularly permuted catalytic polypeptide chains: implications for protein folding and assembly. Protein Sci. 1996;5:1290‐1300. 8819162 10.1002/pro.5560050708 PMC2143468

[132] Viguera AR , Serrano L , Wilmanns M . Different folding transition states may result in the same native structure. Nat Struct Biol. 1996;3:874‐880. 8836105 10.1038/nsb1096-874

[133] Otzen DE , Fersht AR . Folding of circular and permuted chymotrypsin inhibitor 2: retention of the folding nucleus. Biochemistry. 1998;37:8139‐8146. 9609709 10.1021/bi980250g

[134] Hennecke J , Sebbel P , Glockshuber R . Random circular permutation of DsbA reveals segments that are essential for protein folding and stability. J Mol Biol. 1999;286:1197‐1215. 10047491 10.1006/jmbi.1998.2531

[135] Parmeggiani F , Huang Po‐S . Designing repeat proteins: a modular approach to protein design. Curr Opin Struct Biol. 2017;45:116‐123. 28267654 10.1016/j.sbi.2017.02.001

[136] Huang Y‐M , Nayak S , Bystroff C . Quantitative in vivo solubility and reconstitution of truncated circular permutants of green fluorescent protein. Protein Sci. 2011;20:1775‐1780. 21910151 10.1002/pro.735 PMC3267941

[137] Lo W‐C , Lee C‐C , Lee C‐Yu , Lyu P‐C . CPDB: a database of circular permutation in proteins. Nucleic Acids Res. 2009;37:D328‐D332. 18842637 10.1093/nar/gkn679 PMC2686539

[138] Chen C‐C , Huang Yu‐W , Huang H‐C , Lo W‐C , Lyu P‐C . SeqCP: a sequence‐based algorithm for searching circularly permuted proteins. Comput Struct Biotechnol J. 2023;21:185‐201. 36582435 10.1016/j.csbj.2022.11.024 PMC9763678

[139] Cao L , Coventry B , Goreshnik I , et al. Design of protein‐binding proteins from the target structure alone. Nature. 2022;605:551‐560. 35332283 10.1038/s41586-022-04654-9 PMC9117152

[140] Huang Po‐S , Boyken SE , Baker D . The coming of age of de novo protein design. Nature. 2016;537:320‐327. 27629638 10.1038/nature19946

[141] Bannas P , Hambach J , Koch‐Nolte F . Nanobodies and nanobody‐based human heavy chain antibodies as antitumor therapeutics. Front Immunol. 2017;8:1603. 29213270 10.3389/fimmu.2017.01603 PMC5702627

[142] Schilling J , Jost C , Ilie IM , et al. Thermostable designed ankyrin repeat proteins (DARPins) as building blocks for innovative drugs. J Biol Chem. 2022;298:101403. 34793836 10.1016/j.jbc.2021.101403 PMC8683736

[143] Kudla G , Plech M . Lighting up protein design. eLife. 2022;11:e79310. 35588054 10.7554/eLife.79310 PMC9119673

[144] Gonzalez Somermeyer L , Fleiss A , Mishin AS , et al. Heterogeneity of the GFP fitness landscape and data‐driven protein design. eLife. 2022;11. 10.7554/eLife.75842 PMC9119679 35510622

[145] Kondrashov DA , Kondrashov FA . Topological features of rugged fitness landscapes in sequence space. Trends Genet. 2015;31:24‐33. 25438718 10.1016/j.tig.2014.09.009

[146] He L , Tan P , Zhu L , et al. Circularly permuted LOV2 as a modular photoswitch for optogenetic engineering. Nat Chem Biol. 2021;17:915‐923. 33958793 10.1038/s41589-021-00792-9

[147] Kook YH , Lee H , Lee J , et al. AAV‐compatible optogenetic tools for activating endogenous calcium channels in vivo. Mol Brain. 2023;16:73. 37848907 10.1186/s13041-023-01061-7 PMC10583393

[148] Bali B , Lopez De La Morena D , Mittring A , et al. Utility of red‐light ultrafast optogenetic stimulation of the auditory pathway. EMBO Mol Med. 2021;13:e13391. 33960685 10.15252/emmm.202013391 PMC8185542

[149] Renaud N , Geng C , Georgievska S , et al. DeepRank: a deep learning framework for data mining 3D protein‐protein interfaces. Nat Commun. 2021;12:7068. 34862392 10.1038/s41467-021-27396-0 PMC8642403

[150] Gligorijević V , Renfrew PD , Kosciolek T , et al. Structure‐based protein function prediction using graph convolutional networks. Nat Commun. 2021;12:3168. 34039967 10.1038/s41467-021-23303-9 PMC8155034

[151] Jumper J , Evans R , Pritzel A , et al. Highly accurate protein structure prediction with AlphaFold. Nature. 2021;596:583‐589. 34265844 10.1038/s41586-021-03819-2 PMC8371605

[152] Eisenstein M . AI‐enhanced protein design makes proteins that have never existed. Nat Biotechnol. 2023;41:303‐305. 36823357 10.1038/s41587-023-01705-y PMC9949690

[153] Kim DE , Jensen DR , Feldman D , et al. De novo design of small beta barrel proteins. Proc Natl Acad Sci U S A. 2023;120:e2207974120. 36897987 10.1073/pnas.2207974120 PMC10089152

[154] Au HKE , Isalan M , Mielcarek M . Gene therapy advances: a meta‐analysis of AAV usage in clinical settings. Front Med. 2021;8:809118. 10.3389/fmed.2021.809118 PMC8864161 35223884

[155] Shen W , Liu S , Ou Li . rAAV immunogenicity, toxicity, and durability in 255 clinical trials: a meta‐analysis. Front Immunol. 2022;13:1001263. 36389770 10.3389/fimmu.2022.1001263 PMC9647052

[156] Shieh PB , Kuntz NL , Dowling JJ , et al. Safety and efficacy of gene replacement therapy for X‐linked myotubular myopathy (ASPIRO): a multinational, open‐label, dose‐escalation trial. Lancet Neurol. 2023;22:1125‐1139. 37977713 10.1016/S1474-4422(23)00313-7

[157] uniQure . Huntington's disease . uniQure https://www.uniqure.com/programs‐pipeline/huntingtons‐disease

[158] Astellas Pharma . Notice regarding impairment loss for products under development . Astellas Pharma. https://www.astellas.com/en/news/25731

[159] Lv J , Wang H , Cheng X , et al. AAV1‐hOTOF gene therapy for autosomal recessive deafness 9: a single‐arm trial. Lancet. 2024;24:S0140‐6736(23)02874‐X. doi:10.1016/S0140-6736(23)02874-X 38280389

[160] Hoy SM . *Onasemnogene abeparvovec*: first global approval. Drugs. 2019;79:1255‐1262. 31270752 10.1007/s40265-019-01162-5

[161] Kumaran N , Michaelides M , Smith AJ , Ali RR , Bainbridge JWB . Retinal gene therapy. Br Med Bull. 2018;126:13‐25. 29506236 10.1093/bmb/ldy005

[162] Chowdhury EA , Meno‐Tetang G , Chang HY , et al. Current progress and limitations of AAV mediated delivery of protein therapeutic genes and the importance of developing quantitative pharmacokinetic/pharmacodynamic (PK/PD) models. Adv Drug Deliv Rev. 2021;170:214‐237. 33486008 10.1016/j.addr.2021.01.017

[163] Bijlani S , Pang KaM , Sivanandam V , Singh A , Chatterjee S . The role of recombinant AAV in precise genome editing. Front Genome Ed. 2021;3:799722. 35098210 10.3389/fgeed.2021.799722 PMC8793687

[164] Gaudet D , Stroes ES , Méthot J , et al. Long‐term retrospective analysis of gene therapy with *Alipogene tiparvovec* and its effect on lipoprotein lipase deficiency‐induced pancreatitis. Hum Gene Ther. 2016;27:916‐925. 27412455 10.1089/hum.2015.158

[165] Maguire AM , High KA , Auricchio A , et al. Age‐dependent effects of RPE65 gene therapy for leber's congenital amaurosis: a phase 1 dose‐escalation trial. Lancet. 2009;374:1597‐1605. 19854499 10.1016/S0140-6736(09)61836-5 PMC4492302

[166] Konkle BA , Walsh CE , Escobar MA , et al. BAX 335 hemophilia B gene therapy clinical trial results: potential impact of CpG sequences on gene expression. Blood. 2021;137:763‐774. 33067633 10.1182/blood.2019004625 PMC7885820

[167] Leavitt AD , Konkle BA , Stine K , et al. Updated follow‐up of the alta study, a phase 1/2 study of *Giroctocogene fitelparvovec* (SB‐525) gene therapy in adults with severe hemophilia a. Blood. 2020;136:12.

[168] Muhuri M , Levy DI , Schulz M , Mccarty D , Gao G . Durability of transgene expression after rAAV gene therapy. Mol Ther. 2022;30:1364‐1380. 35283274 10.1016/j.ymthe.2022.03.004 PMC9077371

[169] Hinderer C , Katz N , Buza EL , et al. Severe toxicity in nonhuman primates and piglets following high‐dose intravenous administration of an adeno‐associated virus vector expressing human SMN. Hum Gene Ther. 2018;29:285‐298. 29378426 10.1089/hum.2018.015 PMC5865262

[170] Roca C , Motas S , Marcó S , et al. Disease correction by AAV‐mediated gene therapy in a new mouse model of mucopolysaccharidosis type IIID. Hum Mol Genet. 2017;26:1535‐1551. 28334745 10.1093/hmg/ddx058

[171] Mccarty DM , Dirosario J , Gulaid K , Muenzer J , Fu H . Mannitol‐facilitated CNS entry of rAAV2 vector significantly delayed the neurological disease progression in MPS IIIB mice. Gene Ther. 2009;16:1340‐1352. 19587708 10.1038/gt.2009.85 PMC4289609

[172] Wilson JM , Flotte TR . Moving forward after two deaths in a gene therapy trial of myotubular myopathy. Hum Gene Ther. 2020;31:695‐696. 32605399 10.1089/hum.2020.182

[173] Chand DH , Zaidman C , Arya K , et al. Thrombotic microangiopathy following *Onasemnogene abeparvovec* for spinal muscular atrophy: a case series. J Pediatr. 2021;231:265‐268. 33259859 10.1016/j.jpeds.2020.11.054

[174] Pfizer . Pfizer's new phase 1b results of gene therapy in ambulatory boys with duchenne muscular dystrophy (DMD) support advancement into pivotal phase 3 study. Pfizer. https://www.pfizer.com/news/press‐release/press‐release‐detail/pfizers‐new‐phase‐1b‐results‐gene‐therapy‐ambulatory‐boys

[175] Fitzpatrick Z , Leborgne C , Barbon E , et al. Influence of pre‐existing anti‐capsid neutralizing and binding antibodies on AAV vector transduction. Mol Ther Methods Clin Dev. 2018;9:119‐129. 29766022 10.1016/j.omtm.2018.02.003 PMC5948224

[176] Boutin S , Monteilhet V , Veron P , et al. Prevalence of serum IgG and neutralizing factors against adeno‐associated virus (AAV) types 1, 2, 5, 6, 8, and 9 in the healthy population: implications for gene therapy using AAV vectors. Hum Gene Ther. 2010;21:704‐712. 20095819 10.1089/hum.2009.182

[177] Klamroth R , Hayes G , Andreeva T , et al. Global seroprevalence of pre‐existing immunity against AAV5 and other AAV serotypes in people with hemophilia A. Hum Gene Ther. 2022;33:432‐441. 35156839 10.1089/hum.2021.287 PMC9063149

[178] Martino AT , Herzog RW , Anegon I , Adjali O . Measuring immune responses to recombinant AAV gene transfer. Methods Mol Biol. 2011;807:259‐272. 22034034 10.1007/978-1-61779-370-7_11 PMC3593270

[179] Bertin B , Veron P , Leborgne C , et al. Capsid‐specific removal of circulating antibodies to adeno‐associated virus vectors. Sci Rep. 2020;10:864. 31965041 10.1038/s41598-020-57893-z PMC6972890

[180] Weber T . Anti‐AAV antibodies in AAV gene therapy: current challenges and possible solutions. Front Immunol. 2021;12:658399. 33815421 10.3389/fimmu.2021.658399 PMC8010240

